# Current developments and trends in quantum crystallography

**DOI:** 10.1107/S2052520624003421

**Published:** 2024-06-18

**Authors:** Anna Krawczuk, Alessandro Genoni

**Affiliations:** a Institut für Anorganische Chemie, Georg-August-Universität, Tammannstraße 4, Göttingen, 37077, Germany; b Université de Lorraine and CNRS Laboratoire de Physique et Chimie Théoriques 1 Boulevard Arago Metz 57078 France; University of Antwerp, Belgium

**Keywords:** quantum crystallography, multipole model methods, wavefunction-based approaches, quantum chemical topology, charge density-property relationships

## Abstract

Quantum crystallography is an emerging research field of science that has its origin in the early days of quantum physics and modern crystallography when it was almost immediately envisaged that X-ray radiation could be somehow exploited to determine the electron distribution of atoms and molecules. Today it can be seen as a composite research area at the intersection of crystallography, quantum chemistry, solid-state physics, applied mathematics and computer science, with the goal of investigating quantum problems, phenomena and features of the crystalline state. In this article, the state-of-the-art of quantum crystallography will be described by presenting developments and applications of novel techniques that have been introduced in the last 15 years. The focus will be on advances in the framework of multipole model strategies, wavefunction-/density matrix-based approaches and quantum chemical topological techniques. Finally, possible future improvements and expansions in the field will be discussed, also considering new emerging experimental and computational technologies.

## Abstract 2

Recent methodological developments and their applications in quantum crystallography are reviewed, with an eye towards near-future advancements in this research field.

## 1. Introduction

At first sight, it might seem that the term quantum crystallography (QCr) has been recently introduced only to gather electron density-based investigations within the broader domain of crystallography. Nevertheless, the emerging field of quantum crystallography has quite deep roots that date back to the dawn of quantum physics, which was practically the same time when crystallography started being based on X-ray diffraction (Debye, 1915 ▸; Compton, 1915 ▸). As interestingly reported by Macchi (2020 ▸, 2022 ▸) in his complete historical reconstruction of the quantum crystallography origins, the early quantum physicists immediately realized that the high-energy X-ray radiation used to carry out the new crystallographic experiments could be also profitably exploited to ‘visualize’ the distributions of electrons in atoms and molecules. As done by Macchi, in this context it is always enlightening to cite the precursory words by Debye and Compton: ‘*It seems to me that the experimental study of the scattered radiation, in particular from light atoms, should get more attention, since along this way it should be possible to determine the arrangement of the electrons in the atoms*’ (Debye, 1915 ▸); ‘*It is hoped that it will be possible in this manner* [through X-ray diffraction] *to obtain more definite information concerning the distribution of the electrons in the atoms.*’ (Compton, 1915 ▸).

Nowadays, more than a century later, those words by Debye and Compton can be considered as the starting point of quantum crystallography. However, what is quantum crystallography today? A conclusive and inclusive definition of the term has not been given yet and constructive discussions and debates are still in place within the community. In fact, on the one hand, the current definitions of quantum crystallography simply arise from the applications of crystallography in the realm of quantum mechanics (for problems and phenomena in chemistry, physics and materials science) and, conversely, from the applications of quantum mechanics to crystallography (Genoni, Bučinský *et al.*, 2018 ▸). However, on the other hand, for a more complete definition, one should also account for the true quantum effects that appear in the interaction of radiation with matter.

Within this framework, several techniques have been introduced and successfully exploited over the years to investigate the crystalline state from a purely quantum-mechanical point-of-view. Among them, we can certainly include: (i) methods based on the traditional multipole models for the experimental determination of static charge and spin densities (Dawson, 1967 ▸; Kurki-Suonio, 1968 ▸; Stewart, 1969 ▸, 1976 ▸; Hirshfeld, 1971 ▸; Coppens *et al.*, 1971 ▸; Hansen & Coppens, 1978 ▸; Deutsch *et al.*, 2012 ▸, 2014 ▸); (ii) maximum entropy strategies (Sakata & Sato, 1990 ▸; Roversi *et al.*, 1998 ▸; Van Smaalen & Netzel, 2009 ▸) to obtain experimental dynamic charge density distributions; (iii) purely quantum chemistry techniques successfully implemented in popular software for periodic *ab initio* computations [*e.g.CRYSTAL* (Erba *et al.*, 2023 ▸; Dovesi *et al.*, 2022 ▸), *Quantum Espresso* (Giannozzi *et al.*, 2017 ▸; Carnimeo *et al.*, 2023 ▸) or *Wien2K* (Blaha *et al.*, 2020 ▸)]; (iv) all quantum chemical topological strategies for the analysis and interpretation of theoretical or experimental electron densities [*e.g.* the quantum theory of atoms in molecules (QTAIM) (Bader, 1990 ▸) along with the related source function (Bader & Gatti, 1998 ▸; Gatti *et al.*, 2015 ▸) and interacting quantum atom (Blanco *et al.*, 2005 ▸) approaches, and the noncovalent interaction index technique (Johnson *et al.*, 2010 ▸)]; (v) methods characterized by a strong interplay between quantum chemistry and X-ray diffraction measurements (Grabowsky *et al.*, 2017 ▸, 2020 ▸; Genoni & Macchi, 2020 ▸).

**Figure 1:**
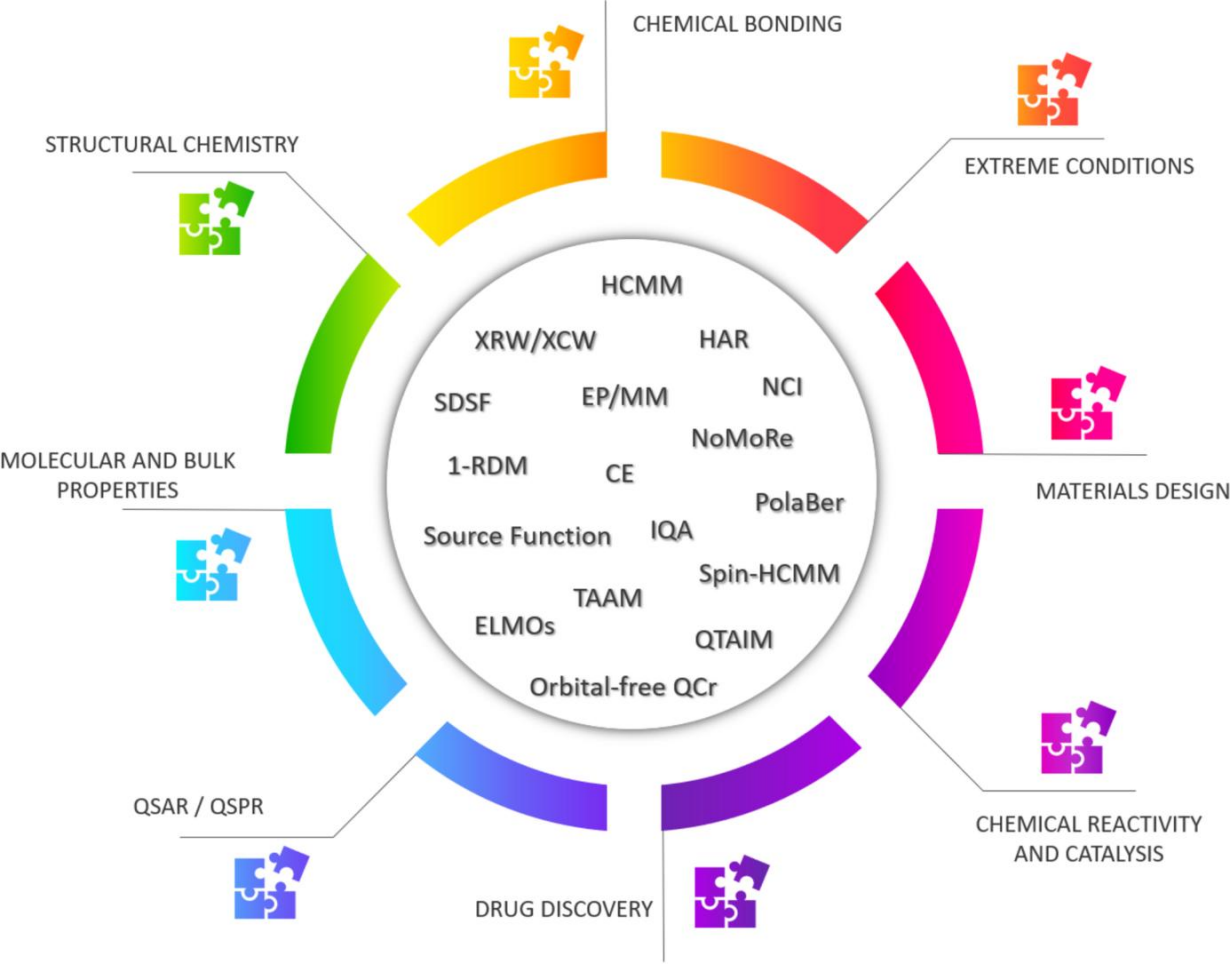
Overview of quantum crystallography methods and areas of their applications. Definition of the acronyms appearing in the figure (in alphabetic order): 1-RDM – one-electron reduced density matrix methods; CE – *Crystal Explorer*-based approaches; ELMOs – extremely localized molecular orbitals; HAR – Hirshfeld atom refinement-based techniques; EP/MM – exact potential/multipole moments method; HCMM – Hansen–Coppens multipole model; IQA – interacting quantum atom approach; NCI – non-covalent interaction technique; NoMoRe – normal mode refinement; *PolaBer* – distributed atomic polarizability approach; QTAIM – quantum theory of atoms in molecules; SDSF - spin density source function; Spin-HCMM: Hansen–Coppens multipole model for spin density refinement; XRW/XCW – X-ray restrained/constrained wavefunction techniques; TAAM – transferable aspherical atom model.

In this article, the progress in the field of quantum crystallography in the last 15 years is reviewed, and a look at possible future directions is also given. To this purpose, the paper is organized as follows: Section 2 deals with recent advances in multipole model methods for charge and spin density refinements; Section 3 is dedicated to new developments in wavefunction- and density-matrix-based approaches; Section 4 focuses on advancements in methods for the topological analyses of electron densities; Section 5 considers studies in which properties have been successfully extracted after the refinement of electron densities or wavefunctions against experimental diffraction data; finally, in Section 6, possible future trends in quantum crystallography are discussed. In Fig. 1 ▸ we have tried to graphically summarize the content of this article and, consequently, the state-of-the-art in quantum crystallography. In the ‘core’ of the figure we have grouped all the techniques that have recently been developed and are often applied in quantum crystallographic studies, while, on the outer edge, we have highlighted the possible domains of applications of the QCr methods.

## 2. Multipole model-based techniques

The multipole model of electron density was proposed and improved over the years by several authors (Dawson, 1967 ▸; Kurki-Suonio, 1968 ▸; Stewart, 1969 ▸, 1976 ▸; Hirshfeld, 1971 ▸; Coppens *et al.*, 1971 ▸) and it was later formalized in the currently most used version by Hansen and Coppens (1978 ▸). Nowadays, it represents a well established approach to determine accurate and detailed electron density distributions of molecules starting from experimental X-ray diffraction data. Unlike the simpler spherical atom model (also called the independent atom model, IAM), the multipole strategy takes into account the anisotropic nature of the atomic electron densities when atoms are involved in chemical bonds by considering both spherical and non-spherical contributions, thus providing a more realistic depiction of chemical interactions. In the Hansen & Coppens formalism (1978 ▸), each atomic density is defined as follows:
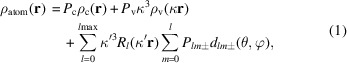
where the most crucial term is the third one, which accounts for the aspherical nature of the valence shell and is modelled *via* the real spherical harmonics 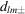 and the associated 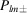 population parameters. The contraction and expansion of the valence shell is described by including the 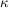 and 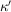 parameters, while the population of the spherical core and valence shells is embodied through the *P*_c_ and *P*_v_ parameters, respectively.

Without any doubt, over the years the multipole model method has made a significant contribution to our understanding of structure–property correlations and has become a stimulus to further improve the description of charge density, particularly in cases where significant disorder is observed or when the access to sufficiently high-resolution X-ray diffraction (XRD) data is limited. One efficient way to overcome these quite common crystallographic drawbacks takes advantage of the fact that the parameters describing the aspherical behaviour of the atomic densities (*i.e.* the pseudo­atoms) are practically identical in chemically related environments and, therefore, transferable. This means that one could expect a reliable estimation of the electron density distribution around a designated atom type, and further around a whole molecule in a crystalline material. In this direction, a proof of concept was given by Brock, Dunitz and Hirshfeld (Brock *et al.*, 1991 ▸), who indeed showed that it was possible to construct a sufficient multipole model of a new molecule with previously defined deformation densities in a series of π-conjugated systems. The idea was picked up rather fast by the QCr community and the first databank of experimentally obtained electron density parameters from high-resolution X-ray diffraction data was introduced in 1995 by Pichon-Pesme *et al.* (1995 ▸), namely the ELMAM (Experimental Library of Multipolar Atom Model) database. Although the databank was initially constructed from amino acids and small peptides, the fast development of diffraction techniques (in particular, X-ray sources, detectors, dedicated beamlines at synchrotron facilities, *etc*.) enabled the collection of ultra-high resolution data for other common organic molecules, which led to the extension of the original database to the ELMAM2 library of pseudoatoms (Jelsch *et al.*, 1998 ▸; Domagała & Jelsch, 2008 ▸; Domagała *et al.*, 2012 ▸). In the absence of experimental data, alternative approaches were also proposed, where the first fiddle was played by quantum mechanical computations. In the University at Buffalo Pseudoatom Databank (UBDB; Volkov *et. al.*, 2004 ▸; Dominiak *et al.*, 2007 ▸), the stored pseudoatoms were obtained from multipole model refinements of theoretical electron densities computed on experimental geometries of small molecules taken from the Cambridge Structural Database (CSD; Allen, 2002 ▸). First and second neighbours of the different pseudoatoms were considered and a spawning procedure was used to ensure close transferability of the aspherical atomic electron densities. Similarly, the invariom database (Dittrich *et al.*, 2004 ▸, 2006 ▸, 2007 ▸), later called generalized invariom database (GID; Dittrich *et al.*, 2013 ▸), was also purely theory-based. In that case, non-spherical scattering factors of the Hansen–Coppens multipole model were derived from densities resulting from *ab initio* calculations performed on theoretically optimized molecular geometries and the associated multipole parameters were obtained for unique model compounds. For further details on the comparison of the above-mentioned databanks, the reader is referred to an overview article by Bąk *et al.* (2011 ▸).

Irrespective of the database employed for the stored multipole parameters, the fundamental objectives remain the same: (i) to be transferred to a chosen crystal structure enabling a more precise modelling of the electron density compared to the standard spherical independent atom model, (ii) to improve the quality of the final model without the necessity of providing high-resolution X-ray diffraction data, (iii) to study materials despite observed small-scale disorder of certain functional groups, and (iv) to enable studies of macromolecular systems such as proteins, in particular to accurately approximate their electrostatic properties. Additionally, all the aforementioned approaches fall under the same umbrella name of transferable aspherical atom model (TAAM) procedure, which in the past decade placed significant emphasis on the precise modelling of bio- and macromolecules. This was indeed the domain in which the most notable advances have been achieved by exploiting the multipole model libraries. First of all, in that context, the abovementioned databases significantly contributed to the accurate prediction of electrostatics and interactions energies. The first significant update of the UBDB databank (Bojarowski *et al.*, 2017 ▸) offered estimations of electrostatic interaction energies in conjunction with the EP/MM (exact potential/multipole moments) method. The EP/MM technique evaluates the exact Coulomb integral in the inner region and combines it with a Buckingham-type multipole moments approximation for long-range interatomic interactions. The quantities obtained by exploiting this approach were of chemical accuracy for most types of observed interactions between the considered model molecules, and the octupole level was proven to be sufficient to obtain the convergence of interactions energies. Moreover, those and following studies (Kumar & Dominiak, 2021 ▸; Budniak *et al.*, 2022 ▸) underlined the importance of charge penetration effects, especially when evaluating protein–ligand complexes. These effects are particularly relevant in situations where molecules are in close proximity, although the aforementioned studies showed that they may also occur at distances twice as large as the equilibrium one. This indicated that they cannot be ignored, especially when weak noncovalent interactions occur in the system. Similar conclusions have been drawn by modelling the electrostatic potential of bio- and drug-like molecules (Kumar *et al.*, 2018 ▸ 2019 ▸; Kumar & Dominiak (2021 ▸); Bojarowski *et. al.*, 2022 ▸; Kulik & Dominiak, 2022 ▸). When reconstructing the electrostatic potential (ESP), one should not only focus on the van der Waals (vdW) and higher surfaces, but also on areas smaller than vdW regions. As aptly pointed out, when a small ligand–protein complex is formed, interactions often occur at distances smaller than the sum of vdW radii and are the ones that determine the affinity and molecular recognition in the binding process. Such a close proximity leads to significant modification in the electrostatic potential and, consequently, the entire volume of a molecule should be considered, including the region between the covalent and van der Waals radii.

**Figure 2:**
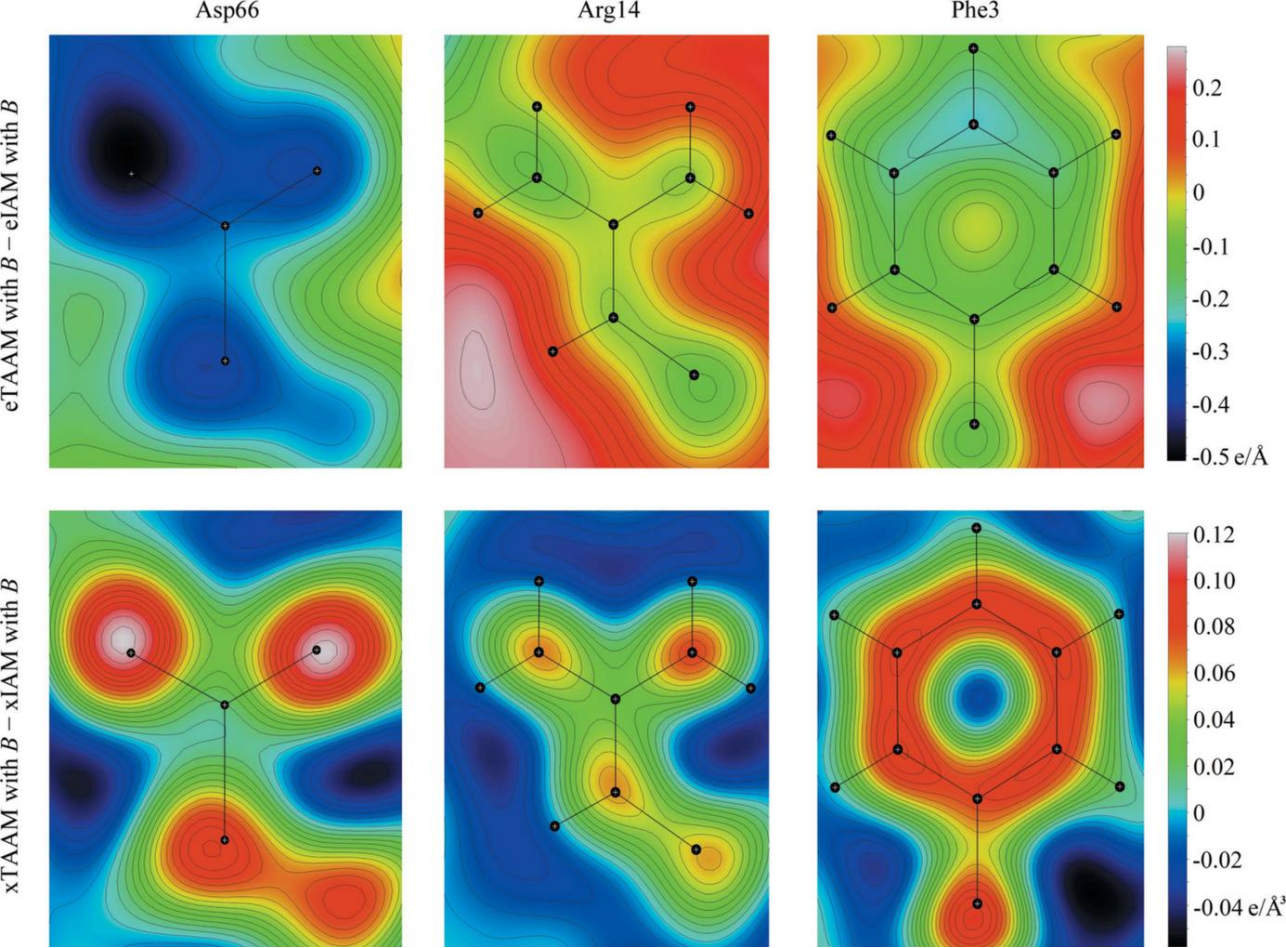
2D *F*_TAAM_ − *F*_IAM_ deformation density maps at 1.8 Å resolution for chosen amino acid side-chains from the lysozyme structure PDB entry 5k7o. The maps take thermal smearing effects into account. Note that the values are given on the absolute scale. Reproduced with permission from Kulik *et al.* (2022 ▸).

Very recently a major update of UBDB was released (Jha *et al.*, 2022 ▸; Rybicka *et al.*, 2022 ▸), including the change of the name to MATTS, standing for Multipolar Atom Types from Theory and Statistical Clustering databank. MATTS has been restructured and expanded to describe multipole parameters of most organic small molecules, peptides, DNA, RNA and common pharmaceutical and biological molecules occurring as ions in a crystalline state. The database currently contains 651 atom types including C, H, N, O, P, S, F, Cl and Br elements and enables the reconstruction of ESPs within the full volume of molecules either by integration of the total electron density in direct space or by Fourier summation in reciprocal space. MATTS also found application in microcrystal electron diffraction (microED), which uses cryo-transmission electron microscopy to collect electron diffraction data from very small micro- and nanocrystals. Dominiak and coworkers made significant progress by exploiting the multipole parameters of the MATTS databank in structural refinements based on microED data, which substantially improved the modelling of density maps obtained via these experiments (see Fig. 2 ▸) (Gruza *et al.*, 2020 ▸; Kulik *et al.*, 2022 ▸). As stated by the authors, there is a notable disproportion between the technological progress in the microED area and the interpretation of the collected data, with the data analysis falling behind. By introducing TAAM in electron crystallography, one includes information on charge redistribution upon chemical bonding (completely neglected when IAM is used), more reliable refinement of ADPs of non-H atoms, and better modelling of electron density-derived properties (in particular, electrostatic potential maps). Undoubtedly, the incorporation of the TAAM technique into the microED structure analysis is a game changer and has a great potential to become a common tool to advance both the data analysis and the reliability of structural information, especially for macromolecules.

In a similar way to its predecessors, MATTS enables the examination of interaction energies, although it does not explicitly incorporate the polarization effects of molecular electron densities caused by interacting neighbouring molecules. In this context, an interesting approach was presented by Guillot, Jelsch and coworkers (Leduc *et al.*, 2019 ▸; Vuković *et al.*, 2021 ▸). The authors combined a database-transferred ELMAM2 multipole electron density with theoretically estimated atomic polarizabilities to address the mutual induction phenomenon and included dipolar functions into transferable pseudoatoms. Such an approach provides a unique opportunity to better simulate electrostatic potentials in the case of interacting molecules and improves the accuracy of the determined intermolecular interaction energies. As a test case, a set of interacting dimers was chosen, for which ELMAM2 provides a full set of transferable multipole parameters. Atomic polarizabilities were determined following a procedure given by Krawczuk & Macchi (2014 ▸), although only atomic polarization components (integrated within QTAIM atomic basins only) were further included. Effects coming from ‘non-atomic’ topological features, described in the work by Krawczuk & Macchi as charge translation components, were fully neglected. Benchmarking against interaction energies obtained with the use of the symmetry-adapted perturbation theory (SAPT) method (Jeziorski *et al.*, 1994 ▸) provided a valuable insight into polarizability-corrected energies. Using the polarizability in the ELMAM2 electron densities produces reliable sets of induction energies, indicating that a substantial portion of the overall induction energy is accounted for by the polarization model. The agreement with the SAPT reference values is remarkably good, thus suggesting that the polarization-corrected ELMAM2 electron densities indeed better describe the electrostatic effects arising from interacting molecules and that further, more extensive and systematic studies should be engaged. Continuing within the area of accurate estimation of interaction energies and electrostatic potentials (not necessarily within the context of pseudoatoms libraries), it is also important to mention the recent contributions made by Volkov and collaborators (Nguyen *et al.*, 2020 ▸; Weatherly *et al.*, 2021 ▸). The research focused on the incorporation of the EP/MM method with the standard Ewald summation (ES) technique, efficiently summing pseudoatom-based atomic multipole moments up to the hexadecapolar level in both direct and reciprocal spaces. The method accounts for the net polarization of the sample (as a result of a net dipole moment) and for short-range electron density penetration effects. In such a way, surface effects arising from varying dielectric properties of the surrounding medium are accounted for. This novel ES/EP/MM approach was successfully used to model: (i) electrostatic interaction energies, (ii) electrostatic potentials, (iii) electric fields (EFs) and (iv) electric field gradients (EFGs) in infinite crystals. In particular, the EP/MM-based ES algorithm proves to be superior to direct-space summations, eliminating the need for continuous monitoring of convergence concerning summation limits, while maintaining a better precision–performance balance. The method is also anticipated to benefit the scientific community in accurately assessing molecule stabilization within the field generated by a periodic arrangement of identical molecules. Ranking intermolecular interactions, particularly in terms of electrostatics, has potential implications for crystal engineering and supramolecular chemistry studies.

Despite numerous advantages of the TAAM approach, it is also important to address its limitation. One must remember that each proposed library is inherently constrained by the number of atom-types that it covers. Furthermore, TAAM fails to accurately account for changes in the global chemical environment when pseudoatoms are transferred to the new target system. Both effects may lead to inaccuracies in the prediction process and naturally restrict the applicability to a limited range of molecular systems. However, by far the most significant limitation of the approach is that all databases rely on an electron density model that is less flexible compared to the fully quantum mechanical ones, particularly when compared to the emerging Hirshfeld atom refinement (HAR) technique. Regarding this, Chodkiewicz *et al.* (2024 ▸) presented preliminary studies on the possibility of constructing a new Hirshfeld atomic density databank (Transferable Hirshfeld Atom Model, THAM), where the quantities are derived from quantum mechanically calculated molecular or ionic wavefunction. Further discussion on THAM will continue in the next section.

**Figure 3:**
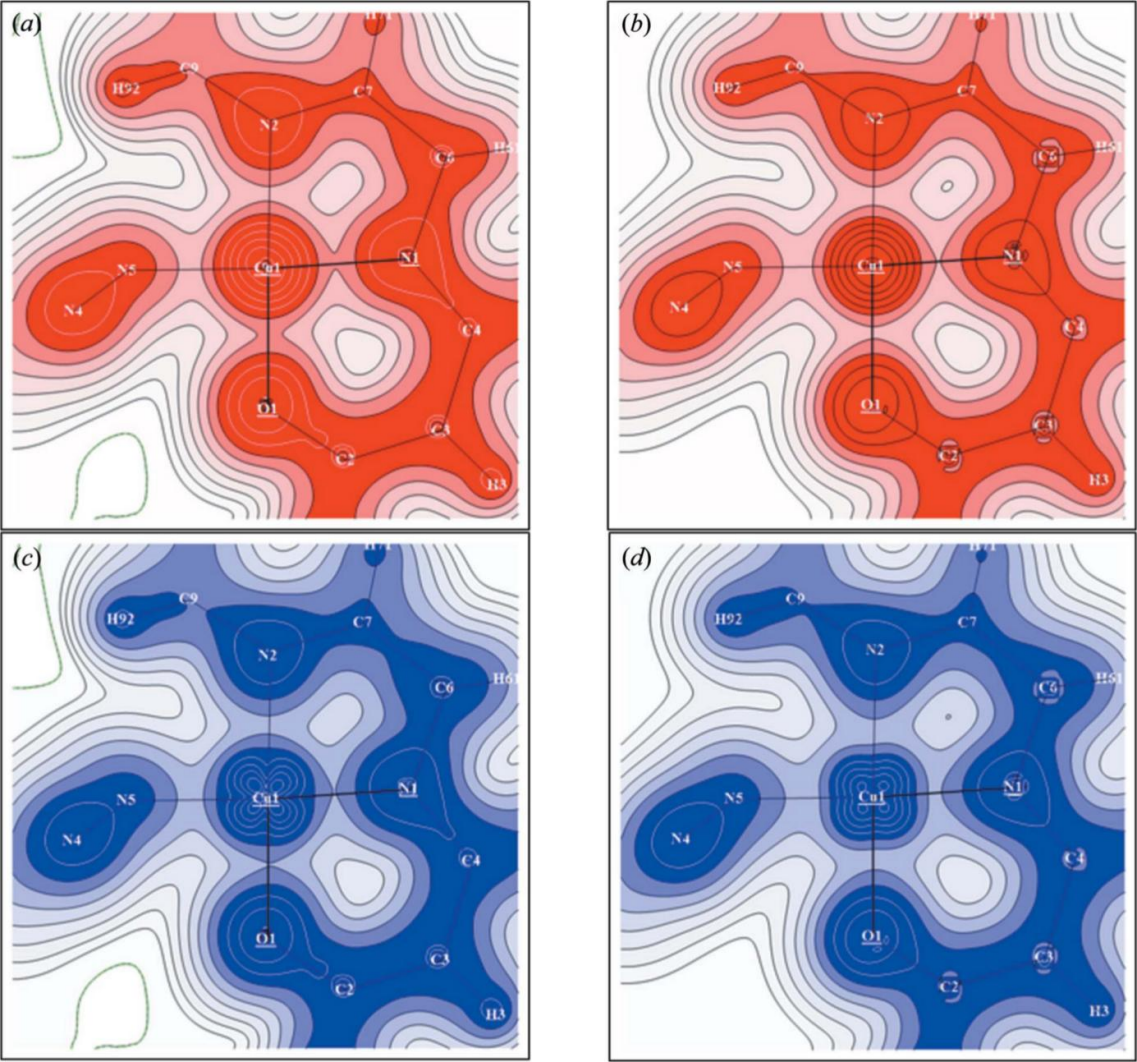
Spin-resolved electron densities obtained from the joint-refinement of X-ray, unpolarized neutron and polarized neutron diffraction data collected for the crystal of the molecular complex Cu_2_*L*_2_(N_3_)_2_ where *L* = 1,1,1-trifluoro-7-[(dimethylamino)-4-methyl-5-aza-3-hepten-2-onato]. Left: (*a*) Experimental spin up (majority) and (*c*) experimental spin down (minority) valence electron densities from joint refinement of the spin-split model. Right: (*b*) Theoretical spin up (majority) and (*d*) theoretical spin down (minority) valence electron densities from *ab initio* quantum computation. The density distributions are represented in the Cu—N1—O1 plane [contours 0.01 × 2^*n*^ e Å^−3^ (*n* = 0–12)]. Reproduced with permission from Deutsch *et al.* (2014 ▸).

When recent developments in the framework of the multipole model-based strategies are considered, one must not forget the efforts made towards the refinement of experimental spin densities. In this context it is worth citing the first joint refinement strategy of charge and spin density distributions, which were performed by introducing a modified version of the Hansen–Coppens multipole model to refine simultaneously X-ray, unpolarized neutron and polarized neutron diffraction data (see Fig. 3 ▸) (Deutsch *et al.*, 2012 ▸, 2014 ▸). More recently, some new improvements of the technique have been also introduced. In particular, an update of the refinement tool MOLLYNX has been reported (Souhassou *et al.*, 2021 ▸), with the purpose of giving a more precise representation of the spin-resolved electron density when high-resolution X-ray, unpolarized neutron and polarized neutron diffraction data are available. The novelty of the approach lies in a dual refinement procedure, which is built upon either the well established version based on the Hansen–Coppens multipole model (MOLLYNX-mult), or the spin-resolved atomic orbital model (MOLLYNX-orb; Kibalin *et al.*, 2021 ▸). While the former provides accurate spin-resolved electron densities, the latter has the advantage of separating one- and two-centre contributions and thus can provide quantitative information on atomic orbital populations. Both models are statistically equivalent to describe electron distributions from experimental data. They can be used for both magnetic (Voufack *et al.*, 2019 ▸) and non-magnetic (Voufack *et al.*, 2017 ▸) crystals, with the only restriction that MOLLYNX-orb is restrained to organic and inorganic materials with small unit cells since two-centre term refinements are very time consuming. Nonetheless, one could imagine that further developments will be presented in the near future. For example, it is possible to envisage hybrid refinements, where inorganic layers will be treated by exploiting the atomic orbital approach, while organic linkers will be tackled through the multipole strategy. Additionally, as already stated by the developers, the next milestone in this area will be ‘*the calculation of wavefunctions dependent properties such as energy, magnetic and first-order optical properties*’ (Souhassou *et al.*, 2021 ▸).

**Figure 4:**
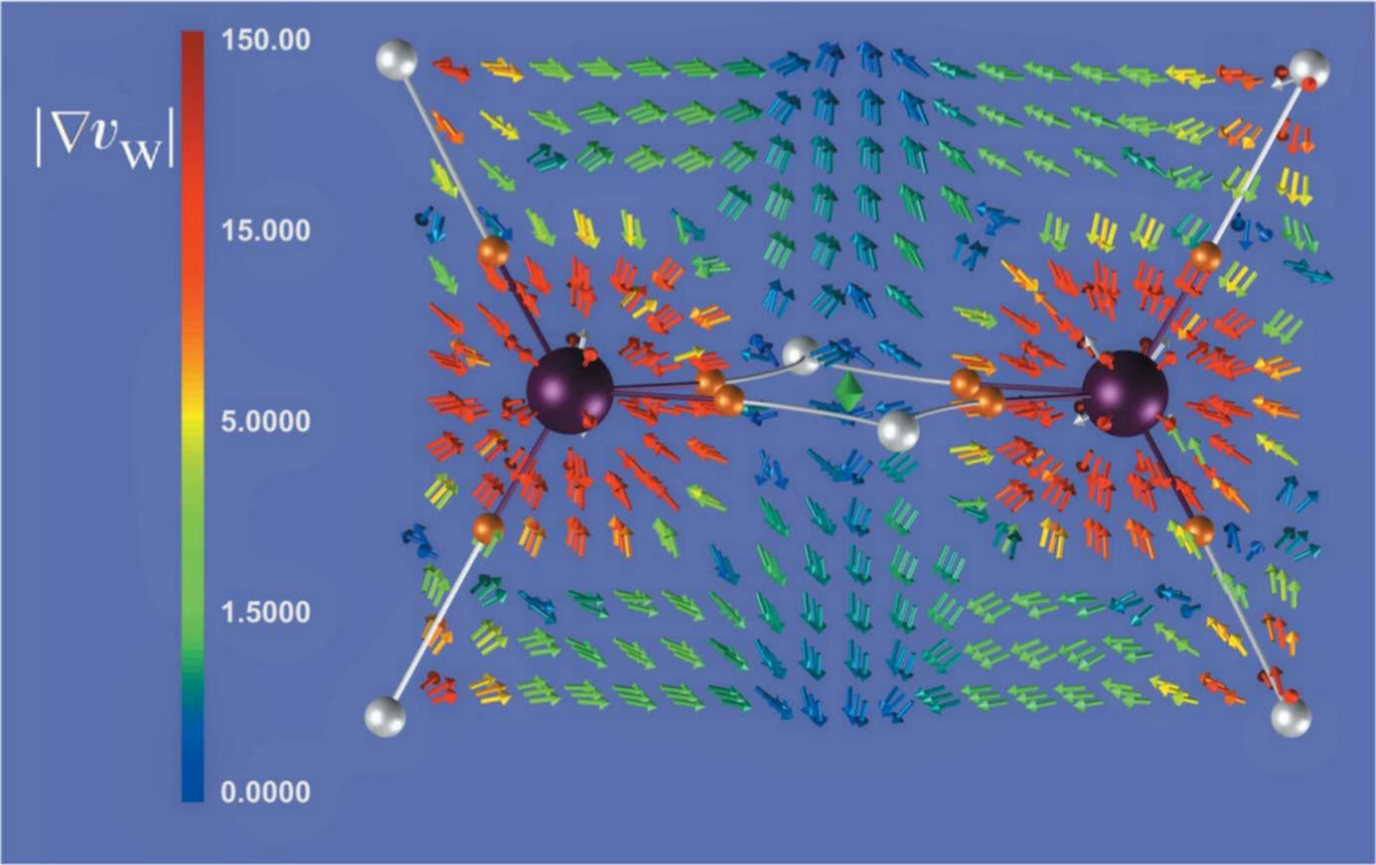
Diborane, B_2_H_6_: 3D vector field of the one-electron von Weizsäcker force in the 0.4 Å layer, superimposed on the bond paths [as obtained with the software *WinXPRO* (Stash & Tsirelson, 2022 ▸)]. The bond critical points are shown as yellow–orange balls, while the ring critical point is depicted as a green cone. The force directions are shown by the arrows, while the force values are indicated by colour (see the scale bar). Reproduced with permission of the International Union of Crystallography from Stash & Tsirelson (2022 ▸).

Among recent developments in multipole model-based techniques it is also worth mentioning the works conducted by Tsirelson and collaborators in proposing orbital-free quantum crystallography (Tsirelson & Stash, 2020 ▸, 2021 ▸; Shteingolts *et al.*, 2021 ▸). In this approach, starting from orbital-free density functional theory (orbital-free DFT) (Wesolowski & Wang, 2013 ▸; Nagy, 2018 ▸; Witt *et al.*, 2018 ▸), the forces acting on electrons in crystals are evaluated by making explicit use of experimental multipole model electron densities. The associated scalar potentials are afterwards topologically analysed (Tsirelson & Stash, 2020 ▸; Shteingolts *et al.*, 2021 ▸). In a more recent version of the approach (Tsirelson & Stash, 2021 ▸), the one-electron Euler equation of orbital-free DFT is rewritten by expressing the static and kinetic potentials in terms of electrostatic, exchange, von Weizsäcker and Pauli components, and the partial electron densities associated with them are also defined through the Poisson equation. This allowed both the decomposition of the total electron density into physically meaningful contributions and a richer interpretation of the information contained in high-resolution X-ray diffraction data, which could be useful for chemical bonding analyses in crystals. The tools of orbital-free quantum crystallography are currently implemented in the software *WinXPRO* (see Fig. 4 ▸) (Stash & Tsirelson, 2022 ▸). Finally, in this context, it is not to be forgotten the bifunctional approach that was recently introduced in orbital-free DFT by Finzel and collaborators (Finzel, 2018*a* ▸, 2020 ▸, 2021*a* ▸,*b* ▸; Finzel & Baranov, 2016 ▸; Finzel & Kohout, 2018 ▸, 2019 ▸) and that could find interesting applications in (orbital-free) quantum crystallography in the near future.

Last but not least, one should also acknowledge the latest works of the Bodensteiner group (Meurer *et al.*, 2022 ▸) on anomalous dispersion corrections, and of Volkov and colleagues (Olukayode *et al.*, 2023 ▸) on the revision of relativistic Dirac–Hartree–Fock X-ray scattering factors of neutral atoms. Although both advancements are not directly related with the Hansen–Coppens multipole model, they provided essential information for the interpretation and analysis of X-ray diffraction patterns, which are of great importance in terms of high-resolution data.

Correcting for anomalous dispersion in X-ray diffraction crystal structure determination involves accounting for inelastic scattering, which is particularly sensitive to radiation energies near the absorption edge of an element. So far, the crystallographic community has relied on tabulated values to correct for those effects, but these estimates overlooked the chemical environment of the element, resulting in poor models with numerous artefacts in residual density maps. Bodensteiner and colleagues introduced a protocol that merges synchrotron multi-wavelength single crystal X-ray diffraction with X-ray absorption spectroscopy to incorporate dispersion parameters *f*′ and *f*′′ in the least-squares refinement. The refined parameters are in good agreement with experimental X-ray absorption spectra and significantly improve the final crystal structure model in terms of better assignment of atomic displacement parameters and electron density, particularly in the vicinity of heavy atoms. The proposed treatment for anomalous dispersion may soon become a common tool for crystal structure determination when heavy elements are present and more reports on the subject are expected.

With the use of a recently developed software (Zatsarinny & Froese Fischer, 2016 ▸), Volkov and others computed fully relativistic Dirac–Hartree–Fock ground-state wavefunctions for atoms with *Z* = 2–118 (He to Og). The calculations employed an extended average level scheme and considered (i) the Breit interaction correction to account for magnetic and retardation effects on the electronic motion, and (ii) the Fermi distribution function to describe the nuclear charge density. The benchmarking of the newly obtained scattering factors against already known databanks, *e.g.* those by Wang *et al.* (1996 ▸), Su & Coppens (1997 ▸) or Macchi & Coppens (2001 ▸), showed a very good agreement for most of the atoms at high resolution (above 0.8 Å^−1^). Such results suggest that the developed database of scattering factors could serve as an additional tool for improving the accuracy and precision of X-ray diffraction data, which could also be of immediate benefit for electron density studies. Further updates on scattering factors of ions are to be published.

## 3. Wavefunction- and density-matrix-based approaches

In this section, the focus will mainly be on the recent advances made in quantum crystallographic methods based on wavefunctions and density matrices (Grabowsky *et al.*, 2017 ▸, 2020 ▸; Genoni & Macchi, 2020 ▸).

Within this context, Hirshfeld atom refinement (HAR) is the strategy that gradually acquired a large popularity even outside the community of quantum crystallographers. Originally introduced by Jayatilaka & Dittrich (2008 ▸), the technique has since been improved over the last 15 years (Capelli *et al.*, 2014 ▸; Woińska *et al.*, 2014 ▸; Fugel, Jayatilaka *et al.*, 2018 ▸; Chodkiewicz *et al.*, 2020 ▸) becoming more and more sound and reliable. At each refinement-iteration the approach usually requires a gas phase quantum chemistry computation to provide an electron density that is afterwards subdivided into atomic contributions according to the Hirshfeld partitioning technique (Hirshfeld, 1977*a* ▸,*b* ▸). The obtained aspherical atoms are then Fourier transformed to get tailor-made aspherical atomic form factors with which the least-squares refinement of the examined crystal structure is performed. The procedure is repeated until convergence is achieved in the structural parameters [*i.e.* atomic positions and anisotropic displacement parameters (ADPs)] by updating the *ad hoc* aspherical atomic form factors at each iteration.

**Figure 5:**
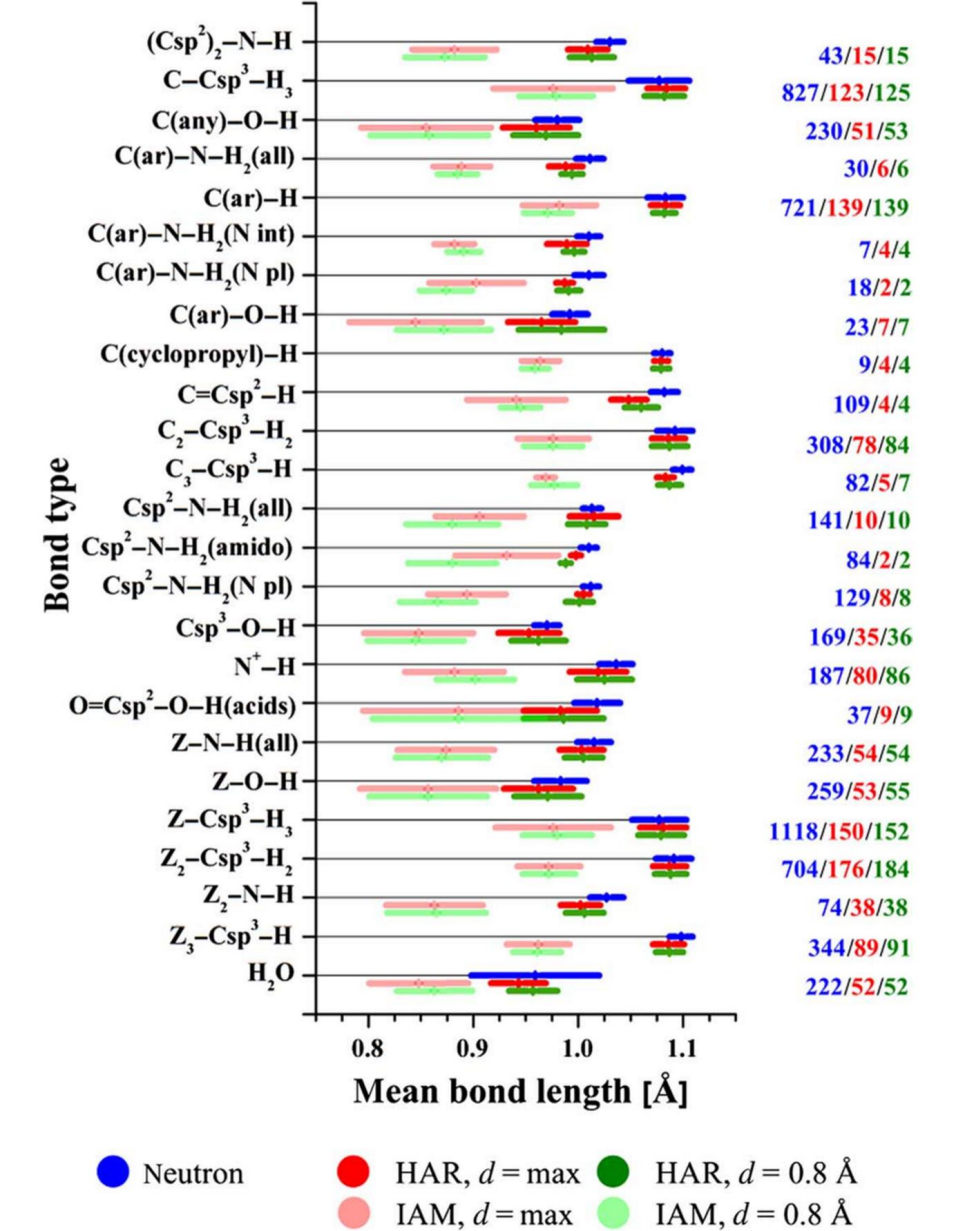
Averaged A—H bond lengths with sample standard deviations obtained from neutron diffraction versus those obtained from X-ray diffraction (HAR and IAM models) at restricted resolution (*d* = 0.8 Å) and with no restriction (*d* = max). Twenty-four bond classes *Z*_*n*_—A—H are taken from Allen & Bruno (2010 ▸) and indicate the atom A bonded to the H atom, and in the case of A = C, the hybridization, and the number of atoms *Z* of any kind with *n* = 1, 2, 3 bonded to the C atom. For cocrystallized water, O—H bond lengths obtained from neutron diffraction using entries in the Cambridge Structural Database (CSD) were averaged. The numbers of observations for all bond types for each refinement method are given in the same colour code in the right-hand column. Reproduced with permission from Woińska *et al.* (2016 ▸).

The turning point that made the method very popular was an investigation (Woińska *et al.*, 2016 ▸) through which it was convincingly proven that, by exploiting only X-ray diffraction data of resolution as low as 0.8 Å, HAR can determine the positions of hydrogen atoms at the same level of precision and accuracy that is usually observed with refinements of neutron diffraction data (see Fig. 5 ▸). This can be undoubtedly considered an important breakthrough in the history of structural refinements. In fact, it was shown for the first time that, provided that a sound aspherical atom model is adopted, hydrogen atoms can be located precisely and accurately by simply performing X-ray diffraction measurements with everyday laboratory diffractometers and without resorting to expensive neutron diffraction experiments that need nuclear reactors or spallation sources. This was also later confirmed by the studies conducted by Köhler *et al.* (2019 ▸) and by Sanjuan-Szklarz *et al.* (2020 ▸). In particular, the former work showed that HAR can indeed provide reliable C—H bonding distances when compared to reference values derived from neutron data, although the corresponding hydrogen ADPs still must be taken with caution.

Another important step that certainly contributed to increase the popularity of HAR was its interface with the freely available *Olex2* refinement software (Dolomanov *et al.*, 2009 ▸; Bourhis *et al.*, 2015 ▸), giving rise to the so-called *NoSpherA2* (non-spherical atoms in *Olex2*) system (Kleemiss *et al.*, 2021 ▸). In this way HAR was combined with both the *Olex2* graphical user interface (GUI) and the *olex2.refine* least-squares engine that is equipped with a complete set of modelling options routinely used in traditional crystal structure refinements. Furthermore, *NoSpherA2* significantly improved the description of core electrons and spin states for compounds containing heavy elements. All these aspects obviously made the use of HAR more user friendly to all those crystallographers that were already familiar with standard structural refinement protocols, and they also allowed the extension of the method to a larger spectrum of chemical systems. Furthermore, one should not overlook the fact that, at approximately the same time, the *DISCaMB* (densities in structural chemistry and molecular biology) library (Chodkiewicz *et al.*, 2018 ▸) was also linked to *Olex2* and the Warsaw branch of HAR was introduced (Chodkiewicz *et al.*, 2020 ▸). This was later applied to several problems (some of them discussed in the following paragraphs), such as determining the positions of hydrogen atoms bonded to heavy metals (Woińska *et al.*, 2021 ▸, 2023 ▸), fragmentation and transferability (Chodkiewicz, Pawlędzio *et al.*, 2022 ▸; Chodkiewicz *et al.*, 2024 ▸), and refinement of ice (Chodkiewicz, Gajda *et al.*, 2022 ▸) and metal–organic framework (Xu *et al.*, 2023 ▸) structures.

**Figure 6:**
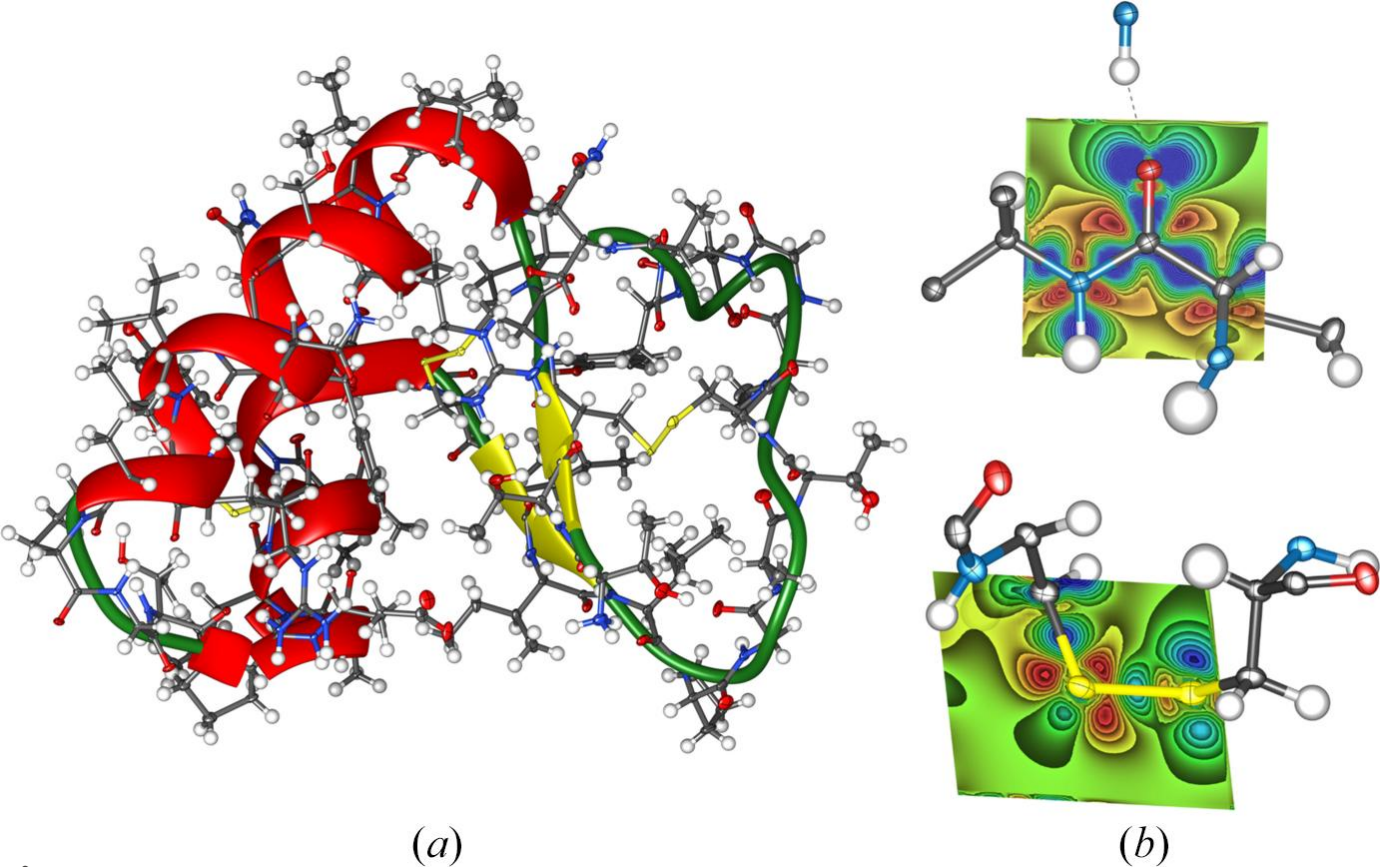
(*a*) Refined protein structure of crambin at 0.54 Å resolution with the HAR-ELMO method, not showing the disordered regions. For clarity, all H atoms are drawn with fixed spheres of 0.3 Å radius. (*b*) Deformation density maps of crambin in a peptide region (C=O in Leu25) and a disulfide bond (between Cys4 and Cys32). Contour interval: 0.05 e Å^−3^, blue = positive, red = negative, green = zero. For (*a*) and (*b*) displacemnt ellipsoids are drawn at the 50% probability level. Reprinted with permission from Malaspina *et al.* (2019 ▸). Copyright 2019 American Chemical Society.

Despite the great advantages associated with HAR, one of the drawbacks of the method is its intrinsic larger computational cost compared to traditional independent atom model (IAM) refinements. This is inextricably associated with the need to perform a quantum chemical computation at each iteration of the procedure, thus preventing a straightforward application of HAR to the refinement of large molecule crystal structures, such as those of biological systems (*e.g.* proteins). To overcome this drawback, two strategies have been proposed: the HAR-extremely localized molecular orbital (HAR-ELMO) method (Malaspina *et al.*, 2019 ▸) and the fragHAR technique (Bergmann *et al.*, 2020 ▸). In the former, HAR was coupled with libraries of extremely localized molecular orbitals (ELMOs) (Meyer *et al.*, 2016*a* ▸,*b* ▸; Meyer & Genoni, 2018 ▸; Wieduwilt, Macetti, Scatena *et al.*, 2021 ▸), which allow instantaneous reconstructions of macromolecular electron densities and consequently bypass the needed quantum chemistry computations. In the latter, HAR was interfaced with the fragmentation technique MFCC (molecular fractionation with conjugate caps; Zhang & Zhang, 2003 ▸) to speed up the underlying theoretical determinations of the electron density. The approaches were successfully used to refine crystal structures of relatively large polypeptides and small proteins (as an example, see Fig. 6 ▸). A significant reduction of the HAR computational cost was observed without affecting the accuracy of the results. Along the lines of the two previous techniques, it is also worthwhile highlighting the fragmentation and transferability scheme applied to HAR by Chodkiewicz and collaborators (Chodkiewicz, Pawlędzio *et al.*, 2022 ▸). This led to the introduction of the concept of transferable Hirshfeld atom model (Chodkiewicz *et al.*, 2024 ▸), thus paving the way for the future development of new libraries of Hirshfeld atom densities that could be fruitfully exploited to perform fast Hirshfeld atom refinements, even of very large systems. The preliminary results indicated that THAM potentially has a computational cost similar to traditional IAM, with structural outcomes comparable to those from HARs without treating the surrounding crystal field.

To improve the results and speed up the convergence of the refinements, the underlying quantum chemical calculations of HAR are generally performed using a surrounding cluster of point charges and dipoles that mimic the crystalline environment. However, this computationally convenient strategy is not generally enough to achieve the desired neutron accuracy for crystals characterized by very strong intermolecular interactions. To overcome this drawback, instead of a cluster of point charges and dipoles, a fully quantum mechanical embedding of frozen ELMOs has been adopted by exploiting the quantum mechanics/extremely localized molecular orbital (QM/ELMO) embedding approach (Macetti & Genoni, 2019 ▸, 2020 ▸, 2021*a* ▸,*b* ▸,*c* ▸; Macetti, Wieduwilt *et al.*, 2020 ▸; Macetti *et al.*, 2021 ▸). The refinements of the xylitol crystal structure performed with the resulting ELMO-embedded HAR strategy (Wieduwilt *et al.*, 2021 ▸) provided element–hydrogen bond distances in optimal agreement with the reference neutron results, while it was not the case for the results of Hirshfeld atom refinements without embedding or with embeddings given by classical charges and dipoles. Along the same line, a further and more sophisticated step towards a fully quantum mechanical embedding in HAR is represented by the first development and application of a HAR version based on periodic *ab initio* calculations. In this case, Ruth *et al.* (2022 ▸) carried out refinements exploiting densities obtained through the projector augmented wave method (Blöchl, 1994 ▸) with periodic boundary conditions. The obtained results in terms of structural parameters were undisputedly superior compared to those of Hirshfeld atom refinements where the crystal environment was completed neglected or described classically.

As one can imagine, a suitable choice of the method and of the basis set for the underlying quantum mechanical computation is crucial in HAR. For this reason, different studies have been conducted to shed light on this aspect. Concerning the basis set, Fugel *et al.* (2018 ▸) considered def2-SVP and cc-pVDZ as adequate, def2-TZVP and cc-PVTZ as excellent, def2-TZVPP and cc-pVQZ as benchmark. Later, Kleemiss *et al.* (2021 ▸) further pointed out that larger and more complete basis sets are generally recommended for accurate treatments of the ADPs, especially for relativistic systems for which one should never use sets of basis functions smaller than triple-zeta. Pertaining to the choice of the quantum mechanical technique, Wieduwilt *et al.* (2020 ▸) carried out a preliminary investigation on the possibility of exploiting correlated wavefunctions. Although further studies may still be necessary to have a complete picture, the results obtained so far seem to indicate that HARs based on post-Hartree–Fock strategies are not significantly better than those based on Hartree–Fock or Density Functional Theory (DFT) calculations. However, more interesting indications came from investigations that aimed to evaluate the impact of the adopted exchange-correlation functionals in DFT-based Hirshfeld atom refinements. For instance, Kleemiss *et al.* (2021 ▸) indicated the use of functional PBE over B3LYP and M06-2X, also suggesting its use along with triple-zeta basis sets as a good trade-off between accuracy and speed. In this context, it is also worth highlighting that Landeros–Rivera *et al.* (2023 ▸) have recently gauged the effect of varying the amount of exact Hartree–Fock exchange in hybrid functionals on the results of HARs performed on crystals of urea and oxalic acid dihydrate. They observed that the element–hydrogen bond lengths systematically increase with the amount of exact exchange, while the displacement ellipsoids become smaller (especially those of hydrogen atoms involved in hydrogen bonds). They concluded by proposing the development of *ad hoc* exchange-correlation functionals for structural refinements.

Another point partially related to the previous one is the first attempt of generalizing the HAR protocol by considering different partitioning methods of the electron density (Chodkiewicz *et al.*, 2020 ▸). This gave rise to the generalized atom refinement (GAR). Only five partitioning strategies have been considered and analysed so far: Hirshfeld, iterative Hirshfeld, iterative stockholder, minimal basis iterative stockholder and Becke. They led to differences in structural parameters comparable to those typically seen with other refinement options. Although the results were only preliminary, the authors suggested that to obtain the best structural parameters from generalized atom refinements (GARs), the choice of the partitioning scheme should be as fundamental as that of the quantum chemistry method and of the basis set (see paragraph above).

To complete this brief overview on recent HAR-based investigations, some interesting applications should be considered, such as the exploitation of HAR to refine crystal structures of organometallic compounds. In this context, Bučinský *et al.* (2016 ▸) exploited the infinite-order-two-component (IOTC) approach to perform the first relativistic HARs, which were later validated by the same authors by exploiting theoretically generated structure factors (Bučinský *et al.*, 2019 ▸). These studies were the starting point for a following series of relativistic HAR-based investigations on an organo-gold(I) compound, which aimed at further validating the relativistic HAR approach (Pawlędzio *et al.*, 2021 ▸), characterizing aurophilic interactions (Pawlędzio *et al.*, 2022*a* ▸) and evaluating the influence of disorder modelling (Pawlędzio *et al.*, 2022*b* ▸). HAR was also successfully used (with and without the inclusion of relativistic effects) to accurately and precisely determine the positions of hydrogen atoms bonded to metal atoms in transition-metal-bound hydride complexes (Woińska *et al.*, 2021 ▸, 2023 ▸). Finally, in the framework of HAR applications, it is worth citing the efforts by Woźniak and collaborators to unravel the different structures of ice (Chodkiewicz, Gajda *et al.*, 2022 ▸).

If on the one hand HAR is certainly the most popular wavefunction-based technique of quantum crystallography, on the other hand the X-ray restrained/constrained wavefunction (XRW/XCW) fitting approach is another perfect example of a strategy belonging to the group of methods described in this section. The technique basically consists of determining a wavefunction that not only minimizes the energy of the investigated system according to the variational principle of quantum chemistry, but that also best fits a set of X-ray diffraction data (Jayatilaka, 1998 ▸; Jayatilaka & Grimwood, 2001 ▸; Grimwood *et al.*, 2003 ▸). Initially known as the X-ray constrained wavefunction (XCW) technique, several authors have recently pointed out that, in its current implementation, the X-ray data do not act as constraints but rather as restraints in the crystallographic sense (Grabowsky *et al.*, 2017 ▸; Ernst *et al.*, 2020 ▸; Macetti *et al.*, 2021 ▸; Genoni, 2022 ▸). For this reason, the approach was renamed as X-ray restrained wavefunction (XRW) method. The latter is the choice that we will adopt throughout the text, but the reader should bear in mind that currently the terms XCW and XRW are frequently used interchangeably in the literature.

**Figure 7:**
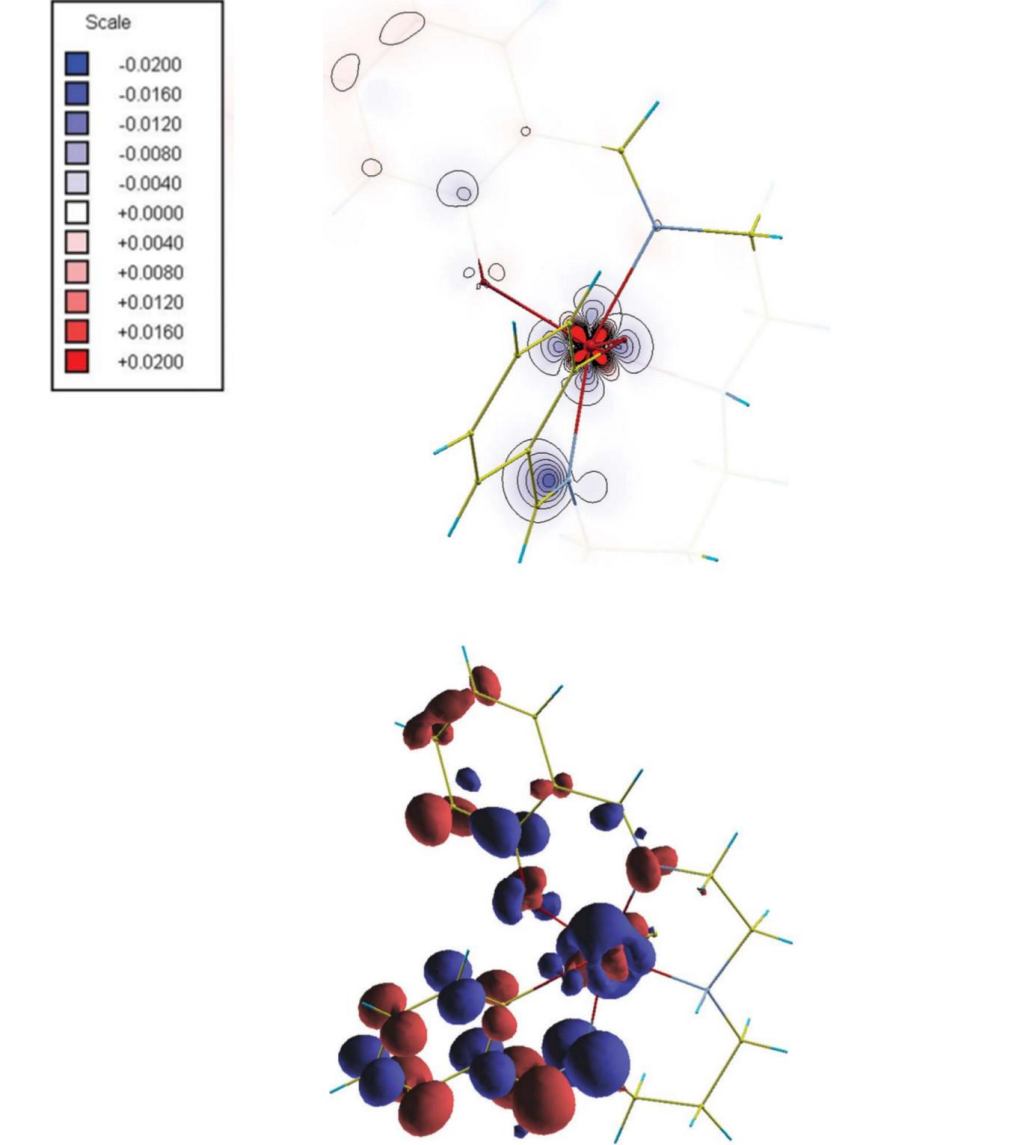
Two- and three-dimensional plots (upper and lower panels, respectively) of the spin density difference ρ_s_(XR − UDKH2) − ρ_s_(UDKH2) [UDKH2 stands for unrestricted second-order Douglas–Kroll–Hess] showing the effects of the wavefunction fitting for the iron coordination compound [Fe(salpet)Cl]. The isovalues for the two-dimensional plot are given in atomic units. The isovalue for the three-dimensional plot is set to 0.01 a.u. Adapted and reproduced with permission of the International Union of Crystallography from Hudák *et al.* (2010 ▸).

The XRW method was initially introduced by Jayatilaka in the framework of the restricted Hartree–Fock formalism (Jayatilaka, 1998 ▸; Jayatilaka & Grimwood, 2001 ▸; Grimwood & Jayatilaka, 2001 ▸; Bytheway *et al.*, 2002 ▸; Bytheway, Grimwood, Figgis *et al.*, 2002 ▸; Grimwood *et al.*, 2003 ▸) and it was gradually extended to other wavefunction *Ansätze* over the years, particularly in the last 15. It was extended to the unrestricted case to treat open-shell systems and coupled with the second-order Douglas–Kroll–Hess approach to include relativistic effects (see Fig. 7 ▸) (Hudák *et al.*, 2010 ▸). Afterwards, it was combined with the Stoll technique (Stoll *et al.*, 1980 ▸; Fornili *et al.*, 2003 ▸) to obtain the so-called X-ray restrained ELMOs (XR-ELMOs) (Genoni, 2013*a* ▸,*b* ▸; Dos Santos *et al.*, 2014 ▸; Genoni & Meyer, 2016 ▸). These XR-ELMOs were also profitably used to embed quantum mechanical calculations of excited states within the framework of QM/ELMO calculations (Macetti *et al.*, 2021 ▸), in the same way as X-ray restrained electron densities at Hartree–Fock level were exploited in frozen density embedding theory (FDET) computations (Ricardi *et al.*, 2020 ▸). First examples of multi-determinant XRW techniques were also recently introduced. One was the X-ray restrained extremely localized molecular orbital-valence bond (XR-ELMO-VB) method, where coefficients associated with preliminarily determined and fixed ELMO wavefunctions are obtained by simultaneously minimizing the electronic energy and maximizing the agreement with experimental X-ray data (Genoni, 2017 ▸). More recently, a more advanced multi-determinant version was proposed by extending the XRW method to the spin-coupled wavefunction *Ansatz* (Genoni, Franchini *et al.*, 2018 ▸; Genoni, Macetti *et al.*, 2019 ▸), in which both the (spin-coupled) orbitals and the determinant coefficients (*i.e.* the spin-coupling coefficients) are to be determined.

In the XRW context, an important place was recently occupied by those studies that aimed to assess the capabilities of the (single-determinant) XRW approach in capturing electron correlation (Genoni, Dos Santos *et al.*, 2017 ▸; Hupf *et al.*, 2023 ▸), polarization (Ernst *et al.*, 2020 ▸; Macetti *et al.*, 2021 ▸; Hupf *et al.*, 2023 ▸) and relativistic effects (Bučinský *et al.*, 2016 ▸, 2019 ▸; Podhorský *et al.*, 2021 ▸). The conducted investigations agree on the fact that the Jayatilaka method is in principle able to get all this information from X-ray diffraction data. This paves the way towards the possible exploitation of the XRW technique for proposing exchange-correlation functionals for DFT calculations based on X-ray data, but provided that further thorough studies and clarifications on the reproducibility (Landeros-Rivera *et al.*, 2021 ▸; Davidson *et al.*, 2022*b* ▸) and the experimental uncertainties (Bürgi & Genoni, 2022 ▸) of the XRW results will be also carried out. Concerning the reproducibility of the XRW results, it is here interesting to cite two recent studies. In one of them, Landeros-Rivera *et al.* (2021 ▸) observed that the XRW results are related to the data treatment, which is hidden in the experimental uncertainties of the structure factor amplitudes used in the XRW computations. In the other investigation, Davidson *et al.* (2022*b* ▸) used 14 different X-ray datasets of the oxalic acid dihydrate crystal to perform XRW calculations, concluding that some systematic effects were consistently observed in the XRW results for each set of data. The authors were able to interpret some of them as electron correlation or polarization effects, but some others were probably related to systematic errors in the experimental measurements.

It is also worth saying a few words on one of the open problems associated with the XRW method: the determination of the parameter λ, which somehow measures the weight with which the experimental X-ray diffraction data are considered in the computations. Interested readers can find a summary of different attempts to establish the λ value in a recent review published on the XRW approach by Davidson *et al.* (2022*a* ▸). However, after the publication of that paper, it was mathematically shown that the longstanding problem of the λ determination could be finally solved by explicitly considering the stationary condition of the original Jayatilaka functional (*i.e.* the electronic energy plus the λ-weighted deviation of the agreement between calculated and experimental X-ray data from a desired value with respect to the λ parameter itself (Genoni, 2022 ▸). This implies a reimplementation of the Jayatilaka approach, where λ will really be a Lagrange multiplier and where the experimental X-ray diffraction data will play the role of partial constraints rather than of restraints (Genoni, 2022 ▸).

In conclusion of this subsection on the XRW method, it is also important to note the recent efforts of profitably combining it with HAR. Although the long-term project is to develop a technique in which HAR and XRW techniques are alternatively iterated until convergence, the current preliminary version of this approach consists of a single HAR followed by an X-ray restrained wavefunction computation, as in the recently proposed X-ray wavefunction refinement (XWR) protocol (Woińska *et al.*, 2017 ▸; Davidson *et al.*, 2022*a* ▸). Interestingly, the XWR procedure was one of the computational techniques exploited to get insights into the mechanism of action of the recently synthesized and proposed sila-ibuprofen (Kleemiss *et al.*, 2020 ▸), a silicon derivative of the common nonsteroidal anti-inflammatory drug ibuprofen.

**Figure 8:**
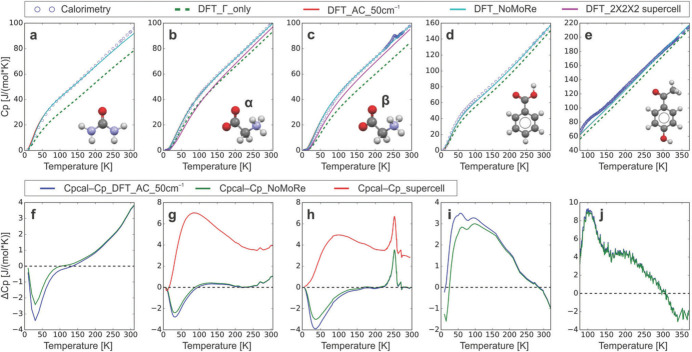
(*a*)–(*e*) Heat capacity for five compounds obtained from calorimetry (blue circles), pure DFT Γ-point calculations (green dashed line), DFT Γ-point calculations with acoustic mode frequencies of 50 cm^−1^ (solid red line) and NoMoRe (solid blue line). (*f*)–(*j*) Difference between heat capacity from calorimetry and DFT Γ-point calculations with acoustic mode frequencies of 50 cm^−1^ (blue line) and NoMoRe (green line). Heat capacity was computed only for temperatures for which the calorimetric data were available (circles). Plots are generated for: (*a*) and (*f*) urea, (*b*) and (*g*) α-glycine, (*c*) and (*h*) β-glycine, (*d*) and (*i*) benzoic acid, (*e*) and (*j*) 4′-hydroxyacetophenone. Reproduced from Hoser *et al.* (2021 ▸) with permission from the Royal Society of Chemistry.

Within the context of wavefunction-based methods of quantum crystallography it is also possible to include the recently developed normal-mode refinement (NoMoRe) approach introduced by Hoser and Madsen (Hoser & Madsen, 2016 ▸, 2017 ▸). The technique starts with a periodic *ab initio* computation that provides normal modes and relative frequencies, which are both exploited to compute the ADPs for all atoms and the structure factors. The statistical agreement between calculated and experimental structure factors is then optimized with respect to the atomic positions, the multipolar parameters (when it is the case), and, above all, with respect to a selected set of vibrational frequencies (generally those corresponding to the low-frequency modes). The procedure is repeated iteratively until convergence is achieved in the statistical agreement. Initially implemented within the IAM framework (Hoser & Madsen, 2016 ▸, 2017 ▸), the strategy has been afterwards interfaced with multipole model refinements (Sovago *et al.*, 2020 ▸) and very recently it has also been coupled with HAR (Woińska *et al.*, 2024 ▸). NoMoRe has been shown to be particularly useful for the evaluation of thermodynamic properties (*e.g.* heat capacities), and these results are generally in good agreement with those obtained through adiabatic calorimetry measurements (see Fig. 8 ▸) (Hoser & Madsen, 2017 ▸; Sovago *et al.*, 2020 ▸; Hoser *et al.*, 2021 ▸). Furthermore, the refinement method was also recently combined with periodic DFT computations to determine the enthalpic and entropic contributions to the free energies of four pyrazinamide polymorphs, thus allowing the prediction of their stability order as a function of temperature and of the corresponding solid-state phase transitions (Hoser *et al.*, 2022 ▸).

**Figure 9:**
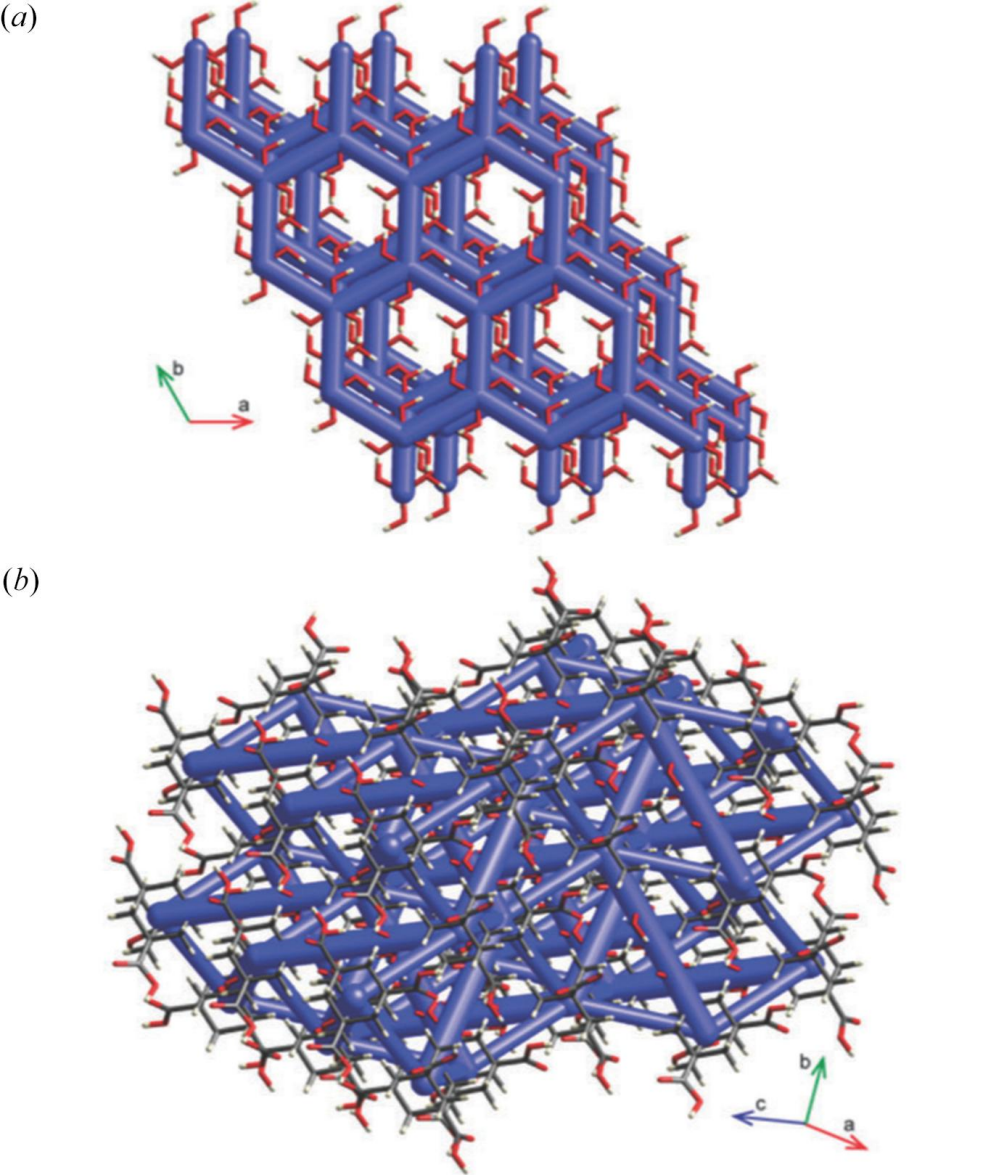
Energy frameworks for the crystal structures of (*a*) orthoboric acid and (*b*) adamantane-1,3,5,7-tetracarboxylic acid. The energy scale factor is 10 and interaction energies with magnitudes less than 15 kJ mol^−1^ have been omitted [see Turner *et al.* (2015 ▸) for details]. Reproduced from Turner *et al.* (2015 ▸) with permission from the Royal Society of Chemistry.

Among the techniques described in this section, a non-negligible place for quantum crystallographic research is also occupied by those wavefunction-based methods that are currently implemented in *Crystal Explorer* (CE), a quite user-friendly and powerful software for the analysis of crystal structures (Spackman *et al.*, 2021 ▸; Spackman, Turner *et al.*, 2021 ▸; Edwards *et al.*, 2017 ▸). CE is useful for: (i) the computation and mapping of properties derived from wavefunctions (*e.g.* electron densities, deformation densities, electrostatic potentials, *etc*.), and particularly the computation and mapping of molecular electrostatic potentials on Hirshfeld surfaces for molecular clusters to get insights into the electrostatic complementarity between adjacent molecules (Spackman *et al.*, 2008 ▸); (ii) the quantification of intermolecular interactions in molecular crystals given by the sum of electrostatic, polarization, dispersion and exchange-repulsion contributions, each of them calibrated against the results of a large number of DFT calculations corrected for basis set superposition error and dispersion (Turner *et al.*, 2014 ▸; Spackman, 2015 ▸; Mackenzie *et al.*, 2017 ▸; Spackman *et al.*, 2023 ▸); (iii) the determination and plotting of the so-called energy frameworks, namely frameworks of cylinders whose widths graphically indicate the strengths of the interactions between neighbouring molecules in order to immediately visualize the topology of interactions in molecular crystals (see Fig. 9 ▸) (Turner *et al.*, 2015 ▸); (iv) the computation of quite accurate lattice energies for molecular crystals (Thomas *et al.*, 2018 ▸; Thomas & Spackman, 2018 ▸). The techniques currently available in CE have already found many applications in many hot topics of solid-state chemistry and physics: (supra)molecular recognition (Shi, Sobolev *et al.*, 2015 ▸; Shi, Thomas *et al.*, 2015 ▸; Dey, Bhandary *et al.*, 2016 ▸; Shi *et al.*, 2016 ▸, 2017 ▸, 2019 ▸; Grosjean *et al.*, 2021 ▸), polymorphism (Dey, Thomas *et al.*, 2016 ▸; Eikeland *et al.*, 2016 ▸; Thomas & Spackman, 2018 ▸; Thomas, Grosjean *et al.*, 2019 ▸), effects of temperature and pressure on crystal structures (Eikeland, Thomsen *et al.*, 2016 ▸, 2017 ▸; Sussardi *et al.*, 2023 ▸), and structure–property relationships (Thomas, Shi *et al.*, 2017 ▸; Karothu *et al.*, 2022 ▸).

Other than methods that use or refine wavefunctions, modern quantum crystallography also comprises techniques that have the goal of determining the one-electron reduced density matrices (1-RDMs) from experimental diffraction data (not necessarily only X-ray diffraction data). Efforts in this direction are certainly those made by Massa, Matta and coworkers, who have recently tried to use and extend to large systems the pioneering approach for density matrix refinements based on the Clinton equations (Massa & Matta, 2018*a* ▸,*b* ▸; Polkosnik *et al.*, 2019 ▸; Matta & Massa, 2022 ▸; Matta *et al.*, 2022 ▸). Furthermore, in this context it is also important to cite the works conducted by Gillet and his collaborators that explored two different research lines: (i) the determination of full 1-RDMs by simultaneously considering X-ray diffraction and directional Compton scattering measurements, and (ii) the reconstruction of spin-resolved 1-RDMs by exploiting both polarized neutron diffraction and magnetic Compton scattering data. Within the first group of techniques, Gillet (2007 ▸) initially extended the Hansen & Coppens multipole model to the refinement of the one-particle reduced density matrices. More recently, in collaboration with De Bruyne, he proposed a more advanced method where the 1-RDMs are expressed in terms of orthogonalized atom-centred basis functions and where the population-matrix elements are determined through a constrained least-squares fitting that implicitly accounts for the *N*-representability conditions on the density matrix by exploiting a semidefinite programming optimization (De Bruyne & Gillet, 2020 ▸). Pertaining to the second research direction, Gillet and coworkers initially performed a preliminary study aiming to reconstruct the spin density of YTiO_3_ both in position and momentum space by using polarized neutron diffraction data and theoretical or experimental incoherent magnetic Compton scattering profiles (Kibalin *et al.*, 2017 ▸; Yan *et al.*, 2017 ▸). Afterwards, they introduced a more sophisticated strategy with the spin-resolved 1-RDM expanded in terms of atom-centred Gaussian basis functions characterized by refinable exponents (Guedidda *et al.*, 2018*a* ▸). In this approach, once the Gaussian exponents are optimized and fixed, the elements of the spin population-matrix are determined through a fitting procedure that maximizes the agreement between calculated quantities and experimental data (namely, magnetic structure factors and magnetic Compton profiles), but always fulfilling the *N*-representability conditions to end up with quantum mechanically rigorous spin-resolved 1-RDMs. The developed technique was initially applied to an artificial magnetic crystal of urea proving the necessity of always simultaneously considering complementary types of experimental data to properly reconstruct the global spin densities of the examined systems (Guedidda *et al.*, 2018*a* ▸). This result was confirmed by the following application of the method to the reconstitution of the spin-resolved one-electron reduced density matrix of YTiO_3_ (Guedidda *et al.*, 2018*b* ▸), which also allowed the investigation of the magnetic properties of the crystal along the Ti–O–Ti bonding directions.

Finally, to conclude this overview of recent advances in wavefunction- and density-matrix-based approaches, one should also be kept in mind the continuous improvements of quantum chemistry software for periodic *ab initio* calculations, which remain fundamental tools for proper comparisons with experimental results in the field of quantum crystallography. In this context, it is worth highlighting that *CRYSTAL* (Erba *et al.*, 2023 ▸; Dovesi *et al.*, 2022 ▸), *Quantum Espresso* (Giannozzi *et al.*, 2017 ▸; Carnimeo *et al.*, 2023 ▸) and *Wien2K* (Blaha *et al.*, 2020 ▸) (namely, the three main software generally used for solid-state computations in quantum crystallography) have all released their new versions in 2023.

## 4. Advances in quantum chemical topological approaches

Quantum chemical topological techniques continue to play a fundamental role in quantum crystallography. In fact, they allow the analysis and the interpretation of the obtained experimental or theoretical electron densities. A prominent place in this role is occupied by the traditional QTAIM (Bader, 1990 ▸), which is often exploited to support not only the multipole model-based charge density studies, but also investigations that use wavefunction-based techniques. Some examples will be mentioned in Section 5. However, if on the one hand the standard QTAIM is well established and ready for use in quantum crystallographic studies, improvements and extensions of the technique have also been devised in recent years. In the following paragraphs, we will present some of them.

An approach strictly related to QTAIM is the source function (SF). Although the original work on the SF for electron density dates back to 1998 (Bader & Gatti, 1998 ▸), in the last decade Gatti and his coworkers devoted to extending and applying the approach to get further insights into the spin density, proposing the so-called spin density source function (SDSF). In their seminal work (Gatti *et al.*, 2015 ▸), the authors showed that the local source function for the spin density provides the total spin density at any point by integrating over all the other points. By integrating the local source function over Bader’s atomic basins, it is possible to determine the contribution of each atom to the spin polarization at any point in real space. Furthermore, one may subdivide these atomic contributions into the magnetic and reaction/relaxation terms, which simplifies the chemical interpretation of the SDSF. As with other more traditional interpretative models that are based on molecular orbitals, the developed SDSF can be profitably used to gain insights into the reasons of spin-polarization effects and to disentangle exchange/pairing mechanisms in magnetic systems. Moreover, being a fully real-space descriptor, the new tool could be extended and used to also analyse spin densities obtained experimentally through multipole model-based (see Section 2) or density matrix-based (see Section 3) approaches, which is not possible for spin density tools based on molecular orbitals. So far, the SDSF technique has been profitably applied to different chemical systems, from the simple water molecule (Gatti *et al.*, 2015 ▸) and *n*-alkyl radicals (Gatti *et al.*, 2016 ▸) to more complicated organo-metallic complexes (Gatti *et al.*, 2017 ▸; Macetti *et al.*, 2018 ▸). Some way related to the work on the SDSF technique, it is worth mentioning that Gatti and coworkers have very recently proposed the spin density topological analysis, thus adding another real-space tool for the interpretation of spin density distributions (Bruno *et al.*, 2020 ▸).

Two other approaches that can be considered as extensions of the original QTAIM are the topological analyses of the Laplacian of the electron density and of the electrostatic potential, both introduced by Espinosa and collaborators to shed light on molecular recognition, assembling and organization. In the first case, the critical points of the Laplacian were instrumental to show that sites of charge concentration and charge depletion in the atomic valence shells are crucial to determine the geometric preferences of molecules in crystals (Bui *et al.*, 2009 ▸; Brezgunova *et al.*, 2012 ▸, 2013 ▸). In the second case, the gradient of the electrostatic potential (namely, the negative of the electric field) allowed the identification of nucleophilic and electrophilic sites in molecules (Mata *et al.*, 2007 ▸). Moreover, from the intersection of the gradient fields of the electron density and of the electrostatic potential, it was possible to get insights into the assembling of anion–anion and cation–cation aggregates (Mata *et al.*, 2012 ▸, 2013 ▸, 2015 ▸; Alkorta *et al.*, 2016 ▸), identifying the spatial regions that contribute to local attractive electrostatic interactions in hydrogen (Mata *et al.*, 2007 ▸) and halogen (Lamberts *et al.*, 2016 ▸) bonds.

Although associable with any type of partitioning scheme of the electron density (and not only with the QTAIM), another quantum chemical topological approach that has recently become more and more important is the interacting quantum atom (IQA) technique (Blanco *et al.*, 2005 ▸; Martín Pendás *et al.*, 2006 ▸, 2007 ▸, 2009 ▸, 2023 ▸; Tiana *et al.*, 2010 ▸). This method decomposes the total energy of a system into self-energy and interatomic contributions. It is based on atomic basins derived from the initial partitioning of the electron density. The interatomic contributions can also be easily expressed as a sum of a classical Coulombic term (associated with the ionicity of the examined system) and of a purely quantum mechanical exchange-correlation term (associated with the covalent nature of the investigated system). Furthermore, by exploiting the subdivision in atomic basins, the IQA decomposition may equally work at the level of group of atoms, which is generally extremely advantageous in many chemical applications. Initially applicable only with Hartree–Fock and post-Hartree–Fock electron densities, the method has been since extended to Kohn–Sham DFT electron distributions by means of a successful partitioning of the DFT exchange-correlation energy (Francisco *et al.*, 2016 ▸; Maxwell *et al.*, 2016 ▸).

In terms of applications, the IQA technique was recently used to shed light on many interesting chemical problems, ranging from reactivity (Jara-Cortés *et al.*, 2018 ▸) to the study of biomolecular systems (Zapata-Acevedo & Popelier, 2022 ▸). However, in the realm of quantum crystallography, the most interesting applications of IQA were those that aimed to get more insights into the description of noncovalent interactions (Popelier, 2022 ▸) and into the chemical bonding analysis. Among the former, as examples, it is worth mentioning that IQA was exploited to rationalize the cooperative effect of hydrogen bonds in water clusters (Guevara-Vela *et al.*, 2013 ▸, 2016 ▸), to understand the role of the exchange-correlation stabilization and of the coulombic contribution in anion–π and halogen-bonding interactions (Foroutan-Nejad *et al.*, 2015 ▸; Niyas *et al.*, 2019 ▸; Jimenéz-Grávalos *et al.*, 2021 ▸), and to unravel the problem related to the stabilizing character of H⋯H contacts (Eskandari & Van Alsenov, 2014 ▸; Matczak, 2016 ▸; Popelier *et al.*, 2018 ▸). Concerning the analysis of chemical bonding, IQA was particularly useful in the investigation of organometallic compounds, which are also frequently studied through other quantum crystallographic approaches. For instance, IQA was exploited to shed light on the electronic properties of metal⋯metal (Werlé *et al.*, 2014 ▸; Caballero-Muñoz *et al.*, 2021 ▸; Lacaze-Dufaure *et al.*, 2022 ▸; Guevara-Vela *et al.*, 2022 ▸), metal–carbene (Sagan *et al.*, 2022 ▸) and metal—carbonyl bonds (Liu *et al.*, 2020 ▸; Van der Maelen, 2020 ▸), and to evaluate the importance of noncovalent interactions in organometallic reactivity (Cukrowski *et al.*, 2014 ▸). However, despite the great usefulness of the IQA strategy to answer many chemical questions (Martín Pendás *et al.*, 2023 ▸), it is also important to note that, in its current implementation, the technique can only work with theoretical electron densities. In the future, it would be desirable to extend the approach to experimental multipole model charge densities and to combine it with XRW computations, although the latter possibility is currently under investigation and reasonable results have already been obtained (Genoni & Martín Pendás, 2024 ▸).

The noncovalent interaction (NCI) index is another very popular quantum chemical topological method in quantum crystallography. This technique was introduced to identify noncovalent interactions in molecular systems (Johnson *et al.*, 2010 ▸; Contreras-García *et al.*, 2011 ▸). To accomplish this task, it uses not only the electron density but also the reduced density gradient. In a nutshell, the NCI-index strategy searches for regions in real space that have both low values of the electron density and low values of the reduced density gradient. It was noticed that these regions are usually observed in correspondence of intra- or inter-molecular noncovalent interactions and their nature [*i.e.* strong (hydrogen-bond) interaction, weak (van der Waals) contact, or steric clash] is easily determined by considering the sign of the second eigenvalue of the electron density Hessian.

**Figure 10:**
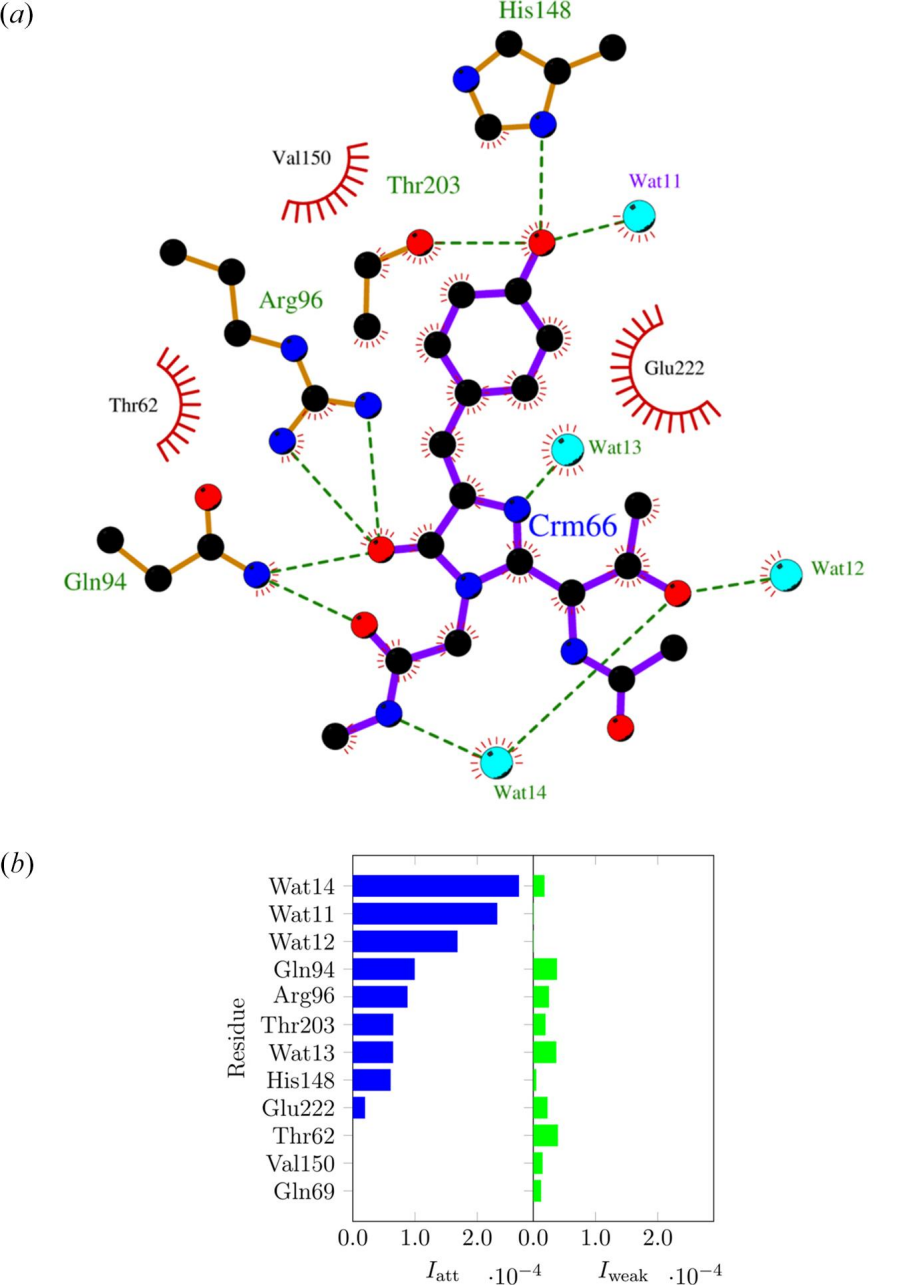
(*a*) LIGPLOT diagram (Wallace *et al.*, 1995 ▸; Laskowski & Swindells, 2011 ▸) graphically showing the interactions between the *p*-hydroxybenzylidene-imidazolinone (pHDBI) chromophore and the surrounding residues of the green fluorescent protein (hydrogen bonds are depicted through green dotted lines, while hydrophobic contacts are represented by means of red arcs with spokes). (*b*) Histograms graphically depicting the strengths of the strongly attractive (*I*_att_) and weak (*I*_weak_) interactions formed by residues of the green fluorescent protein with the pHDBI chromophore in the binding pocket, as resulting from the NCI-QM/ELMO integral analysis with basis set cc-pVDZ. Reprinted with permission from Wieduwilt *et al.* (2023 ▸). Copyright 2023 American Chemical Society.

The NCI index method initially provided only qualitative pictures of noncovalent interactions in molecular systems by exploiting electron densities and reduced density gradients resulting from *ab initio* theoretical calculations or from the superposition of spherically averaged atomic electron densities (especially when dealing with large biomolecules). However, in the last decade, the method has been largely improved by considering different aspects. Among many, it is worth highlighting: (i) its extension through the NCI-Milano version to also consider experimental electron densities resulting from multipole model or maximum entropy refinements (Saleh *et al.*, 2012 ▸, 2013 ▸); (ii) the coupling with the ELMO libraries to perform NCI-index analyses based on quantum mechanically rigorous electron density distributions also in the case of large biomolecular systems (Arias-Olivares *et al.*, 2019 ▸); (iii) the introduction of more advanced variants of the approach to also obtain quantitative results from the NCI-index computations (see Fig. 10 ▸) (Peccati, 2020 ▸; Boto *et al.*, 2020 ▸; Wieduwilt *et al.*, 2023 ▸); (iv) and the creation of a user-friendly webserver to facilitate the analyses of noncovalent interactions in large systems of biological interest (Novoa *et al.*, 2023 ▸). Based on the current state-of-the-art, today the NCI-index approach can be considered as a quite mature technique and more and more applications to quantum crystallographic problems are expected in the next years.

**Figure 11:**
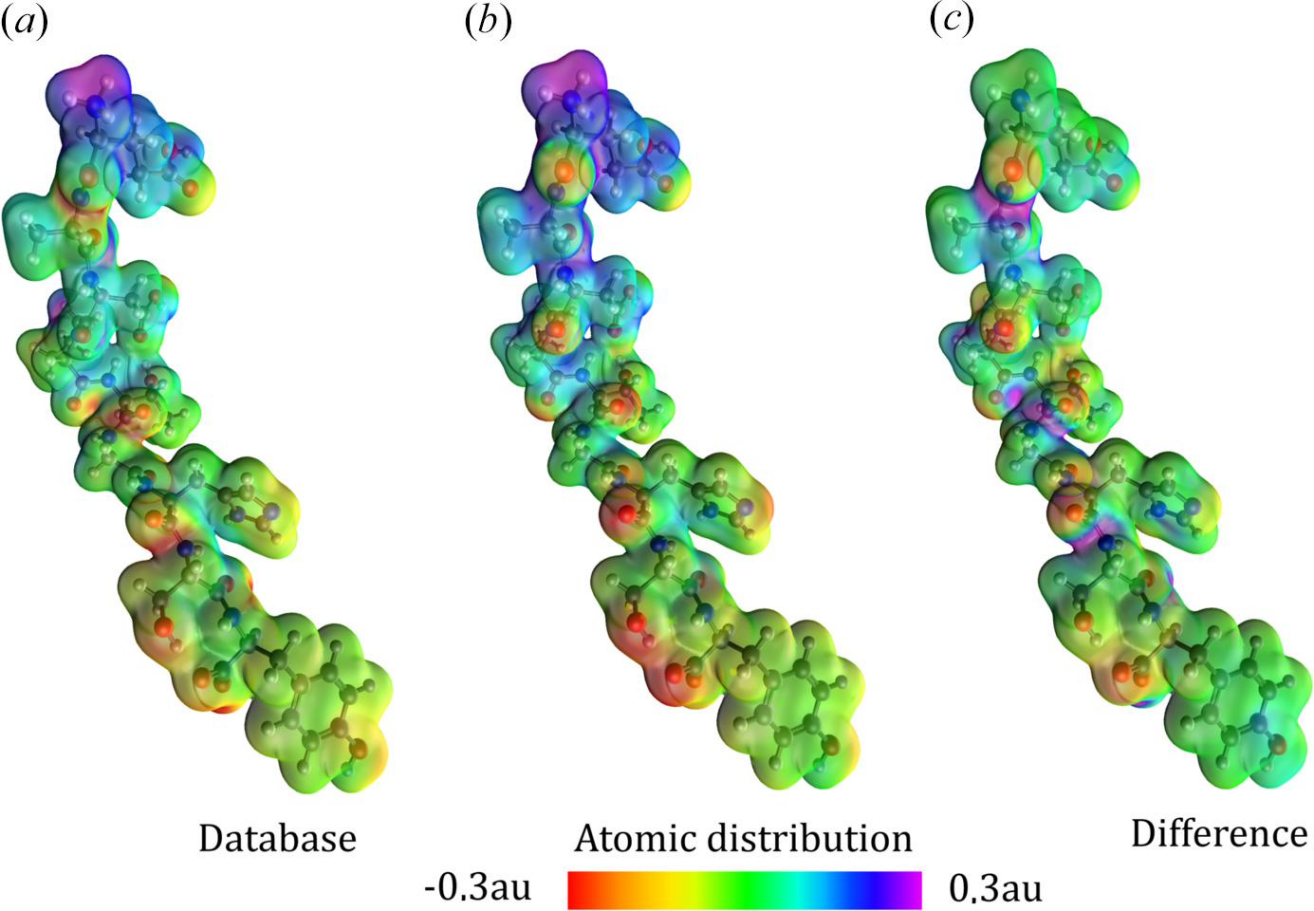
Electrostatic potential maps reconstructed from group dipole moments (database) compared to exact electrostatic potential (atomic distribution) obtained via quantum chemical calculations. Isodensity surface is drawn at 0.01 a.u. level. Reproduced from Ligorio *et al.* (2022 ▸) with permission from the from the PCCP Owner Societies.

Another QTAIM-based strategy that provides valuable insights into the behaviour of a material in response to external influences is the distributed atomic polarizability approach implemented in the *PolaBer* software (Krawczuk *et al.*, 2014 ▸). In brief, *PolaBer* calculates atomic polarizability tensors as numerical derivatives of the atomic dipole moment with respect to the applied electric field. The method is exact as long as the field perturbation is sufficiently small to ensure a linear response (for example, 0.005 atomic units or less). *PolaBer* has proven effective in: (i) determining electron density polarization effects upon the formation of weak non-covalent interactions (Krawczuk & Macchi, 2014 ▸; Dos Santos & Macchi, 2016 ▸), (ii) establishing bond polarizability terms that can be attributed to the concept of hard/soft charge- and orbital-controlled reactions (Macchi & Krawczuk, 2015 ▸; Macchi *et al.*, 2018 ▸) and (iii) identifying the origins of refractive indices of molecular crystals in terms of functional group contributions (Ernst *et al.*, 2016 ▸). Additionally, studies on amino acids and other organic molecules showed remarkable transferability of functional group polarizabilities (Dos Santos *et al.*, 2015 ▸), which gave rise to the group polarizability database (Ernst *et al.*, 2019 ▸). Initially, the databank was designed for fast-screening of molecular crystals towards efficient linear optical properties. However, very soon after, it became apparent that the stored dipole moments and polarizabilities of common functional groups could also be used for larger systems (*e.g.* macromolecules) to predict optoelectronic and electric-response properties, including electrostatic potential (Ligorio *et al.*, 2022 ▸, 2023 ▸; see Fig. 11 ▸). Jabluszewska *et al.* (2020 ▸) showed that by relying only on group dipolar terms it is possible to calculate dipolar electrostatic potentials, which are good estimators of the total electric potentials of molecules. Such a simplified approach, which makes use of functional-group electrostatic potentials (GEP) and distributed polarizabilities (GDP), enables an in-depth analysis of the correlation between structural features and a build-up of molecular properties. The efficiency and accuracy of the database for biomolecules, including macromolecules, were benchmarked against quantum chemical calculations (Ligorio *et al.*, 2022 ▸; Rodrigues *et al.*, 2022 ▸). Aiming at the simulation of condensed phase properties, the dipole interaction model (DIM) was additionally implemented into the polarizability database (Ligorio *et al.*, 2020 ▸, 2021 ▸). It consists of using gas phase dipole moments and polarizabilities, either derived from building blocks or molecular quantities, to estimate the effects of the chemical medium. Therefore, it became possible to predict condensed phase properties, by either explicitly adding a solvent or applying symmetry operations, and to quantify crystal field effects with a reasonable accuracy. Further developments of the polarizability databank are also expected, particularly: (i) its expansion with new entries, such as porphyrin rings, metal ions, sugars, *etc.,* (ii) the inclusion of alternative partitioning schemes other than QTAIM and (iii) testing database entries against polarizable force field methods.

In this section, it is also worth mentioning the continuous efforts in proposing descriptors to analyse chemical bonding, such as the *C_p_* functional introduced by Kohout and collaborators (Wagner & Kohout, 2011 ▸; Finzel *et al.*, 2012 ▸) and based on an inhomogeneity measure of the electron density. For the optimal parameter 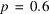, the performances of the new functional were compared with those of the related ELI-D (Kohout *et al.*, 2004 ▸, 2005 ▸, 2008 ▸). It was observed that the two bond descriptors provide a similar topology in the inner-shell and lone-pair regions, but their descriptions deviate significantly in the bonding regions and provide different topologies. However, it is very interesting to observe that, unlike ELI-D, the newer *C_p_* functional can be directly applied to experimental electron densities. Therefore, by exploiting the more recent bonding descriptor, one could in principle obtain binding signatures that do not depend on the theoretical method adopted for the computation, but that are intrinsically associated with the experimental observations.

Finally, to conclude this overview on recent advances in quantum chemical topological approaches, it is interesting to cite some recent efforts to combine QTAIM with machine learning, which might constitute an interesting near-future-perspective for this research field. Examples in this direction are the machine-learnt force field FFLUX developed by Popelier and collaborators (Burn & Popelier, 2020 ▸, 2022 ▸, 2023 ▸; Isamura & Popelier, 2023 ▸), and the NNMAIMGUI code, which was proposed by Gallegos & Martín Pendás (2023 ▸) as an improvement of the previous NNMAIMQ model (Gallegos *et al.*, 2022 ▸) for quick and reliable determinations of partial charges.

## 5. Properties from refined charge densities and wavefunctions

Quantum crystallography does not just propose new methods for the sake of development. In fact, the devised quantum crystallographic strategies are introduced to enhance our ability to explore and understand fundamental properties of matter, leading to advancements in various scientific disciplines and to applications that impact fields ranging from chemistry and physics to materials science and medicine. In this section, we will briefly summarize the latest reports on the use of the aforementioned QCr techniques towards a better comprehension of structure–property correlation. However, for a very detailed discussion on the use of the electron density and of the Hansen–Coppens multipole model in materials science, the reader is referred to an excellent review recently published by Tolborg & Iversen (2019 ▸).

**Figure 12:**
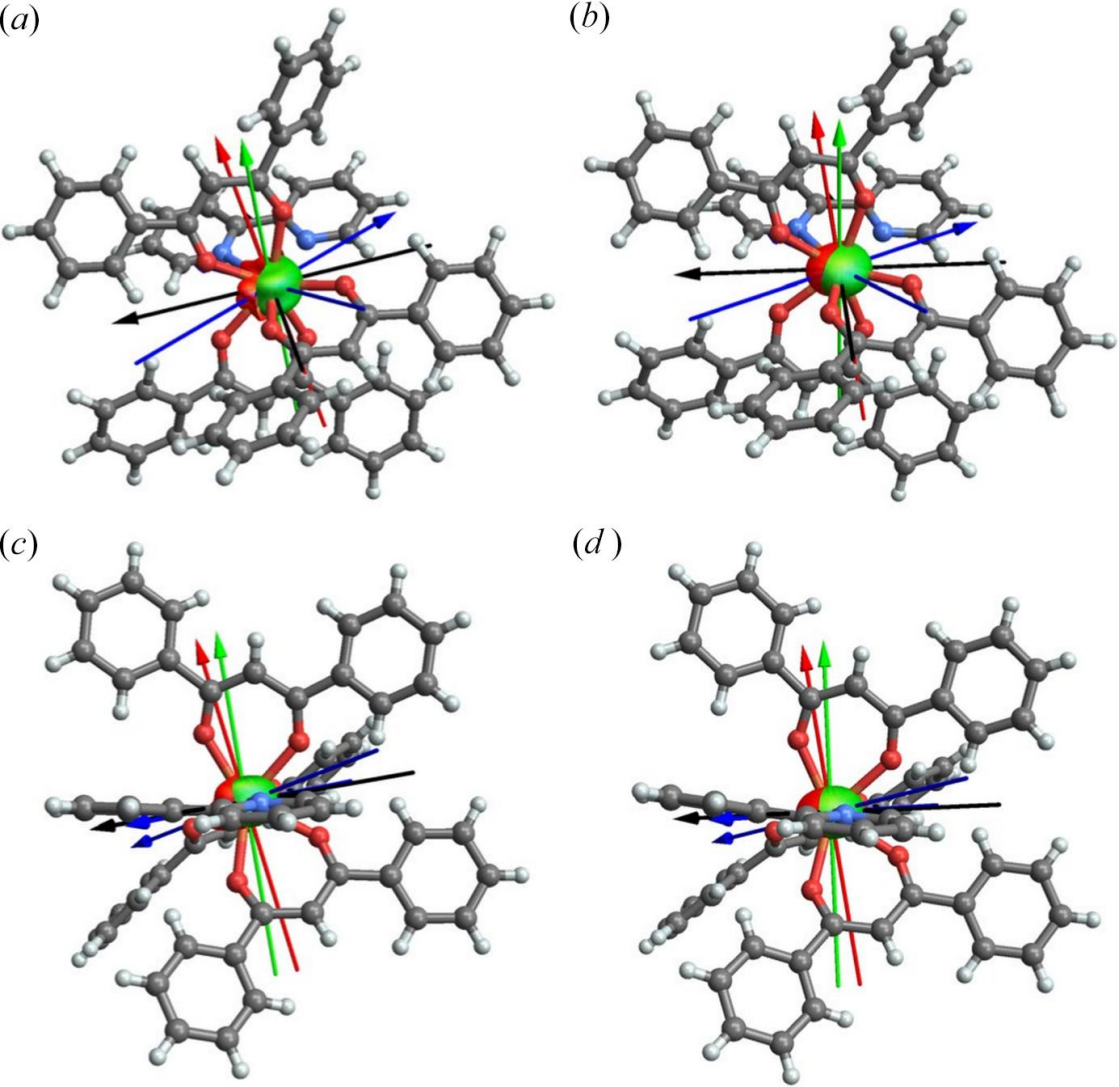
Electron density isosurfaces and unique magnetic axes for two polymorphs of the complex Dy(dbm)_3_(bpy) [with dbm = dibenzoylmethanoate and bpy = 2,2′-bipyridine]. The polymorphs are indicated in this caption as **1A** and **1B**. (*a*)–(*d*) Overlay plots of the molecular structures, showing experimental (red) and *ab initio* (green) 4*f*-electron density, and experimental ellipsoid principal axes represented by red and blue arrows. The red arrows indicate the shortest axis (oblate direction) whereas the two blue arrows indicate the two longer axes. Also shown are the *ab initio* magnetic axes of the ground doublet, with green arrows showing the easy axis and black arrows showing the perpendicular magnetic axes with quasi-zero *g*-values. The isosurfaces have been calculated including all 28 multipoles for **1A** (*a*) and **1B** (*c*) (see (Gao *et al.*, 2020 ▸) for details), and in the ellipsoidal (monopole + quadrupoles) approximation for **1A** (*b*) and **1B** (*d*), as described in Gao *et al.* (2020 ▸) by equations (1a) and (1b) (experimental) and equation (4) (*ab initio*). Grey, carbon; white, hydrogen; blue, nitrogen; red, oxygen. Reproduced from Gao *et al.* (2020 ▸) with permission from Springer Nature.

Among all recent studies that tried to extract meaningful properties from multipole model-based refinements of the electron density, investigations that deserve a particular mention are those conducted by Overgaard and coworkers on single-molecule magnets (SMMs) (Craven *et al.*, 2018 ▸; Thomsen *et al.*, 2019 ▸; Gao *et al.*, 2020 ▸; Damgaard-Møller, Krause, Lassen *et al.*, 2020 ▸; Damgaard-Møller, Krause, Tolborg *et al.*, 2020 ▸; Klahn *et al.*, 2021 ▸; Gupta *et al.*, 2023 ▸). For instance, it is worth highlighting a breakthrough paper where it was reported for the first time the experimental refinement of the aspherical distribution of the 4*f* electrons in two polymorphs of a dysprosium molecular complex and the consequent determination of the associated magnetic anisotropy axes (see Fig. 12 ▸) (Gao *et al.*, 2020 ▸). In this framework, it is also worth citing the multipole model study on the electron distribution for a cobalt(II) complex characterized by a distorted tetrahedron geometry around the metal cation (Damgaard-Møller, Krause, Tolborg *et al.*, 2020 ▸). In this case, the authors observed an aspherical 3d electron density around the metal centre that can be directly associated with the magnetic anisotropy of the investigated molecular magnet. Furthermore, the refined multipole model parameters along with plausible expressions for the spin-orbit coupling wavefunction allowed an estimation of the zero-field splitting (ZFS), which is strictly related to the magnetic anisotropy of the system and determines the barrier height for magnetic relaxation. The obtained values for the zero-field splitting were in very good agreement with a value previously obtained through near-infrared spectroscopy.

**Figure 13:**
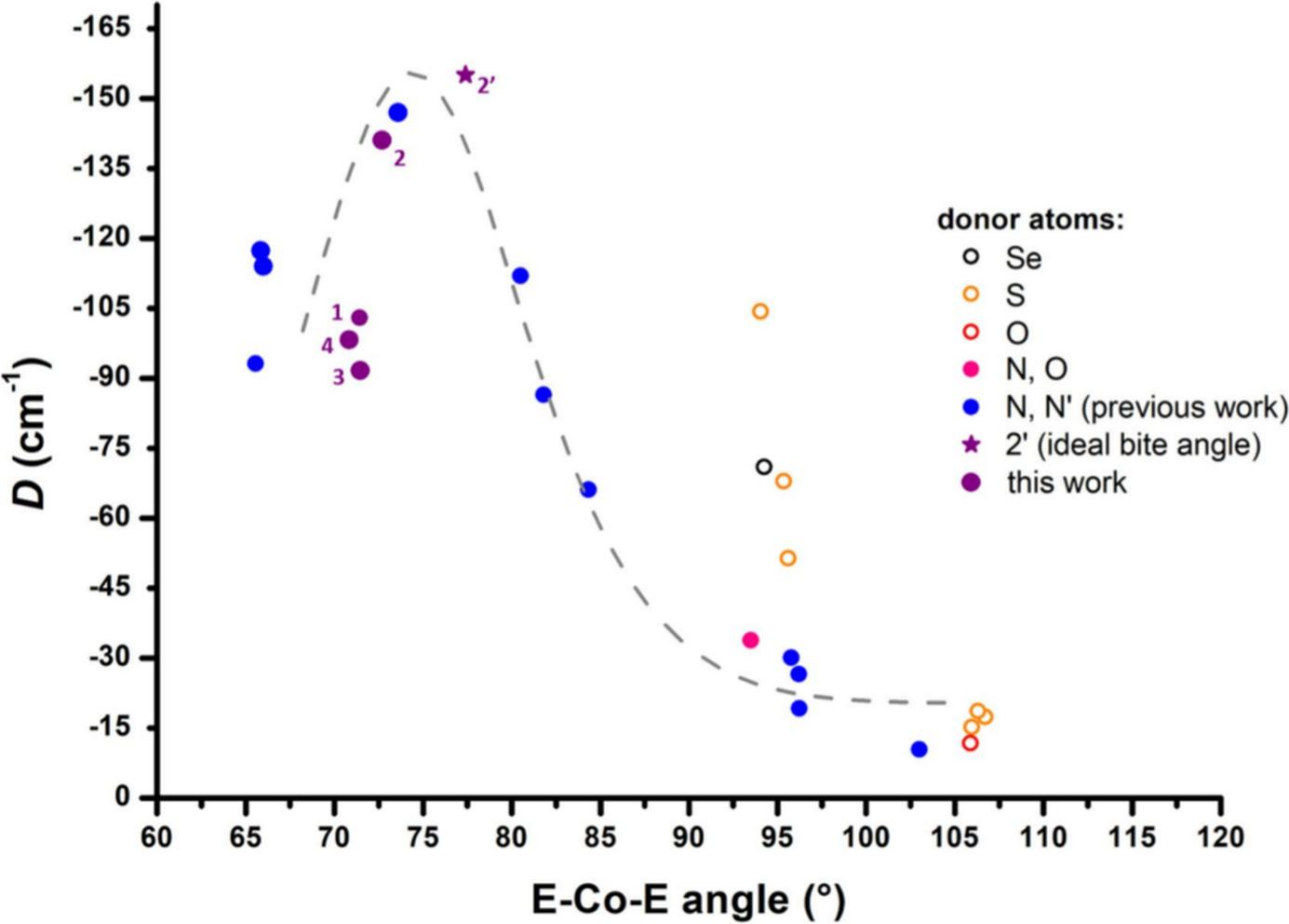
Dependency of the ZFS parameter *D* on the *E*—Co—*E* bite angle in distorted tetrahedral [Co*E*_4_]-SMMs. The grey dashed line is a fit to the [CoN_4_] data points. Reproduced with permission from Legendre, Damgaard-Møller *et al.* (2021 ▸).

While remaining in the field of single-molecule magnets, it is also worth mentioning the latest works carried out by Stalke and collaborators (Legendre, Damgaard-Møller *et al.*, 2021 ▸; Legendre, Lüert *et al.*, 2021 ▸; Jung, Legendre *et al.*, 2021 ▸). In some of the reported SMM complexes, in particular distorted tetrahedral cobalt-containing compounds, the authors observed a colossal magnetic anisotropy that was accompanied by extremely acute bite angles. An unconventional *d*-orbital splitting was uncovered, highlighting the presence of an optimal N—Co—N angle. Those results challenged the conventional belief that achieving a linear C_∞_ geometry through additional *D*_2d_ geometry distortion leads to superior SMM properties. The theoretical assessment suggested that the optimum geometry for [CoN_4_]-SMMs, achievable by magnetic anisotropy maximization, involves a distorted tetrahedron with an ideal bite angle ranging from 76° to 78° (see Fig. 13 ▸).

Stalke and his collaborators also maintain a strong track record in employing the Hansen–Coppens multipole model to comprehend bonding scenarios in diverse organic and inorganic compounds and to correlate the findings with chemical reactivity. For example, experimental and theoretical studies of organolithium compounds (Münch *et al.*, 2020 ▸) confirmed that the Li—C and Li—N bonds show similar characteristics concerning electrostatic and orbital interactions, especially with increasing aggregation. This provided an empirical support for the recent assumption of a predominantly ionic C–Li contact. The rise in ionic character in smaller aggregates facilitates the inclusion of chloride in these structures, aligning with earlier observations in solution experiments where mixed aggregates influenced the reactivity. Another charge density-based study (Jung, Münch *et al.*, 2021 ▸) on H_2_S(N*t*Bu)_4_ marked the first instance of achieving a valence isoelectronic imido analog to sulfuric acid H_2_SO_4_. The topological analysis of the electron density revealed strongly polarized single bonds in both amido S—N(H) and imido S—N bonds. The Laplacian analysis also unveiled a non-symmetrical distribution of the VSCCs (valence-shell charge concentration) around the nitrogen atoms and a tilted one towards the sulfur atom, thus providing an explanation for the observed high bond ellipticities. The inherent polarizability of the entire SN_4_ unit offers adaptability to diverse electronic requirements, showcasing potential applications in the formation of single molecule magnets. In another study (Keil *et al.*, 2021 ▸), Stalke and coworkers provided experimental evidence on the existence of a 3*c*–4*e* bond in a symmetric trichlorine monoanion. QCr analyses provided insights into the impact of the crystalline environment on the structure of [Cl_3_]^−^ anions and the gradual transition from asymmetric to symmetric compounds when compared with Cl_2_ and the Cl^−^ anion.

In the realm of charge density studies, the noteworthy contributions of Iversen and collaborators stand out prominently. Renowned as a leader in leveraging the electron density for discerning correlations between structural features of crystalline materials and various physical properties (*e.g.* magnetic, optical, thermal, *etc.*), the group has also pioneered the use of powder X-ray diffraction data for the accurate determination of electron density distributions of high-symmetry inorganic materials (Svendsen *et al.*, 2010 ▸; Tolborg *et al.*, 2017 ▸). In that area, it is worth mentioning the latest paper on the deconvolution of electron density and thermal motion in diamond at various temperatures (Beyer *et al.*, 2023 ▸). High-resolution powder diffraction data in the temperature range of 100–1000 K were collected with the use of the SPring-8 synchrotron facility. The data analysis indicated that the thermal motion of diamond predominantly displays harmonic and isotropic characteristics, while the topological analysis of the electron density revealed consistent density and Laplacian values at bond critical points (BCPs) irrespective of temperature fluctuations. Those findings imply that the electron density remains unaffected by temperature variations, validating the effective deconvolution of thermal motion from static electron density. Recently the use of the powder diffraction technique for electron density analyses was also extended to simple organic crystalline materials (Svane *et al.*, 2021 ▸), such as urea (space group 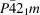) and xylitol (space group 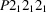). These studies showed that through a technological development joint with a rational treatment of experimental structure factors and a careful multipole refinement, it is possible to obtain reasonable final models of the electron density, comparable to those resulting from single-crystal X-ray diffraction experiments.

**Figure 14:**
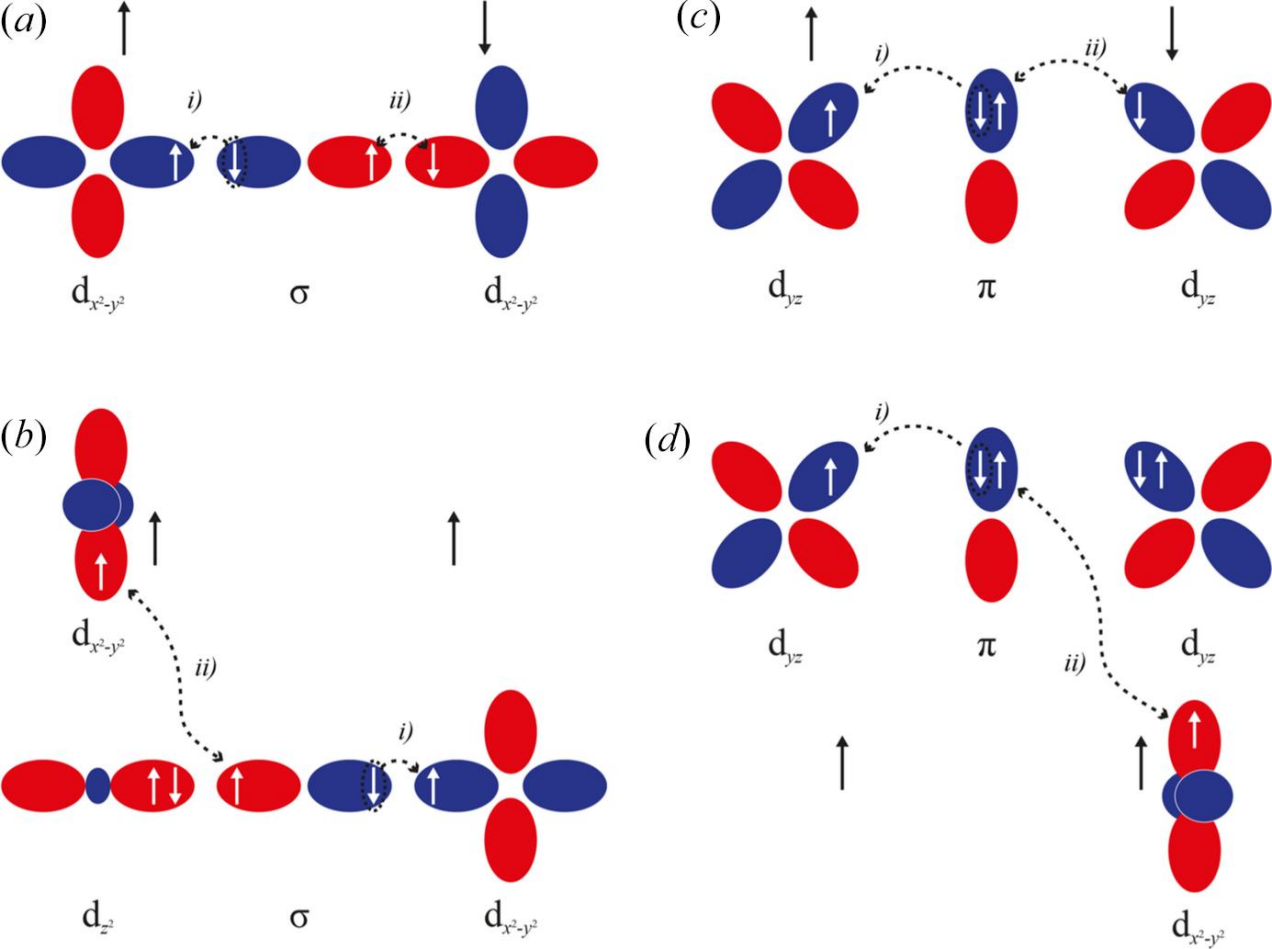
Possible super-exchange mechanisms in Co-formate following Anderson’s mechanism: (i) a virtual transition from ligand orbital to metal orbital adhering to Pauli’s exclusion principle and on-site Hund’s rules followed by (ii) direct exchange between electron left on ligand and the other metal orbital. Black arrows represent the metal site’s overall spin magnetic moment as a result of super-exchange, white arrows are single electrons, single-arrowed dashed lines represent virtual transitions, double-arrowed dashed lines represent exchange, and the delocalized ligand orbitals are represented as *p*-orbitals exhibiting the necessary symmetry. When the ligand orbital is non-orthogonal to a double occupied orbital, an additional single occupied orbital on that metal site is drawn translated vertically. (*a*) Case 1 facilitated through a σ-interaction. (*b*) Case 2 facilitated though a σ-interaction. (*c*) Case 1 facilitated through a π-interaction. (*d*) Case 2 facilitated through a π-interaction. Cases described in main text of Grønbech *et al.* (2023 ▸). Reproduced from Grønbech *et al.* (2023 ▸) with permission from the Royal Society of Chemistry.

In other recent studies, this time using high-resolution single-crystal X-ray diffraction data, Iversen and collaborators focused on structure–property correlations, in terms of magnetic, thermochromic and mechanical properties. In one of them (Grønbech *et al.*, 2023 ▸), the authors aimed at explaining the mechanism of super-exchange in a magnetic Co-formate coordination polymer (see Fig. 14 ▸). Via experimental charge density analysis supported by theoretical modelling, it was possible to assign a magnetic order phenomenon to the super-exchange mechanism between metal sites and formate ligand, where the *d*-populations of the former couple with the σ or π-orbitals of the latter. The Co-formate coordination polymer exhibits a ferromagnetic coupling between the metal centres through a π-facilitated super-exchange, while other compounds (containing Mn, Fe, and Ni) lose the ferromagnetic M(1)–M(2) super-exchange due to altered interaction pathways based on electron count variations. Subsequently, in a paper on the thermochromic properties of the diphenyl diselenide (dpdSe) and diphenyl ditelluride (dpdTe) crystals (Thomas *et al.*, 2023 ▸), topological analyses of the electron density revealed that the change of colour of a material upon temperature variation primarily arises from subtle changes in the torsional vibrational model around the very dynamic Se—Se and Te—Te bonds. Interestingly, intermolecular interactions have only very minor contribution to the property, thus the phenomenon is suspected to originate from a molecular-level vibronic coupling, rather than from a crystal packing-specific electron-phonon coupling. Further studies on a series of similar compounds are expected to confirm this rather unusual hypothesis. Finally, in the paper by Sarkar *et al.* (2022 ▸), the mechanical stability of a coordination polymer has been challenged by means of charge density and high-pressure crystallography investigations. It has been shown that the absence of directional ionicity in metal–ligand bonds facilitates angular distortions in the crystal framework under pressure. This may potentially cause the collapse of the long-range periodicity and may lead to a mechanically induced glass formation. Such a correlation between the nature of chemical bonding and the ability to obtain glass-forming materials may be of immediate benefit in optics, photonics and electronics.

In the context of structure–property correlation investigations it is also worth mentioning some recent updates on the elucidation of (non-)linear optical properties of molecular crystals, particularly in the areas of optical anisotropy of fluorescent materials (Gryl *et al.*, 2020 ▸) and of second harmonic generation (SHG; Wojnarska *et al.*, 2019 ▸, 2021 ▸). In the first case, the authors considered an organic crystal that exhibit substantial optical anisotropy of both absorption and fluorescence. The topological analysis of the electron density, supported by experimental property measurements, showed that the observed maximum effect along (001) is associated with the direction perpendicular to the plane of chromophores, which are connected in a head-to-tail manner through weak dispersive interactions. This unique phenomenon contrasts with the typical quenching of fluorescence caused by the presence of π⋯π interactions. Thus, it has been postulated that the observed optical anisotropy corresponds to the alignment of molecular transition dipole moments induced by a particular molecular self-assembly. In addition, the investigation also underlined the importance of precise measurements of a physical property and its direction-dependence. Upon altering the crystal orientation, the response of the material may differ significantly, and any structure–property correlation should be carefully thought through. Concerning the optical anisotropy of SHG (Wojnarska *et al.*, 2019 ▸, 2021 ▸), the researchers have shown how quantum crystallography tools can facilitate the design of polar crystalline materials that will exhibit second-order nonlinear optical (NLO) properties. To obtain an effective NLO material, the following conditions should be fulfilled: (i) significant molecular hyperpolarizability, and (ii) polar architecture of the crystal, which can be enforced by the presence of certain intermolecular interactions. Indeed, the topological analysis of the electron density along with simultaneous measurements of birefringence and SHG suggested that the presence of intermediate hydrogen bonds (*i.e.* hydrogen bonds between a closed-shell and shared character) in a certain crystallographic direction favours a non-zero dipole moment and a polar arrangement of the molecules. This in turn results in a match of the refractive index of the incident light with that of the second harmonic, and a prominent SHG response can be observed.

**Figure 15:**
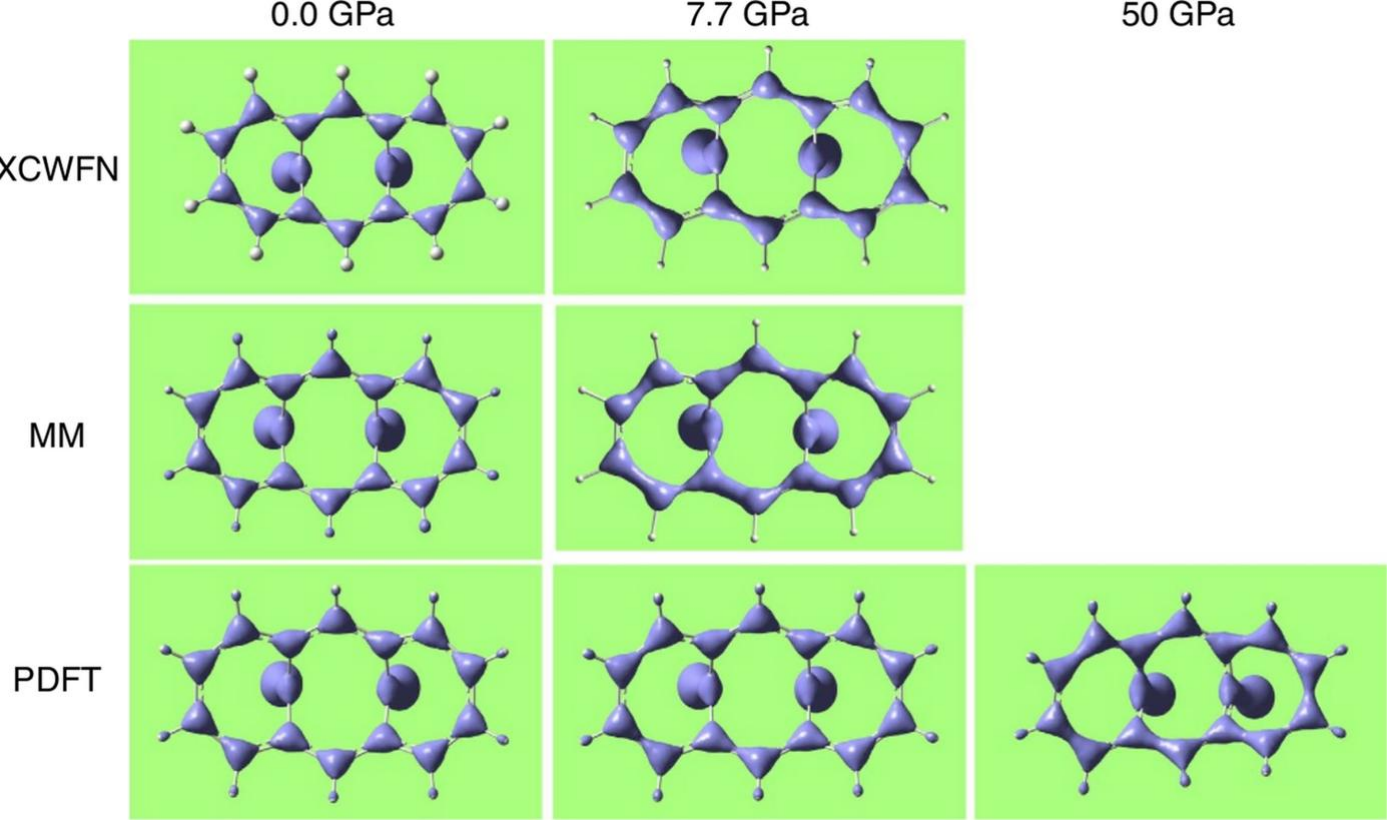
Electron density distribution of BCA. Plots are shown at various pressures and from various sources: PDFT indicates periodic DFT calculations at B3LYP level of theory; XCWFN indicate X-ray constrained (restrained) wavefunction calculations at Hartree–Fock level that used experimentally measured X-ray diffraction data as constraints (restraints); MM is for electron densities derived from multipolar expansion, with coefficients refined against experimentally measured X-ray diffraction intensities. Experimental data at ambient pressure are taken from Destro & Merati (1995 ▸), collected at 19 K; the 7.7 GPa data are from Casati *et al.* (2016 ▸). Reprinted with permission from Casati *et al.* (2016 ▸).

Finally, in reviewing recent noteworthy charge density investigations, one should also highlight a pioneering work conducted by Macchi and coworkers (Casati *et al.*, 2016 ▸), who carried out a very accurate electron density study at high-pressure on bis-carbonyl annulene (BCA). In this case, by exploiting the Hansen–Coppens multipole model along with the XRW approach in its original version (see Fig. 15 ▸) and in the XR-ELMO-VB form (Casati *et al.*, 2017 ▸), the authors were able to unequivocally prove a partial suppression of the BCA aromaticity as pressure increases. In our opinion, this investigation represents a reference for any future electron density study at high pressure.

Naturally, more examples of excellent exploitation of the Hansen–Coppens multipole model and of the topological analysis of electron density in chemistry and materials science are available in the literature. Yet, it is quite impossible to mention and describe all of them in detail in this review. For this reason, we encourage the reader to seek out further excellent scientific reports in the area of chemical bonding (*e.g.* Agnarelli *et al.*, 2023 ▸; Armbrüster *et al.*, 2023 ▸; Fronc *et al.*, 2023 ▸; Hermann *et al.*, 2021 ▸; Vosegaard *et al.*, 2022 ▸; Graw *et al.*, 2023 ▸; Ruth *et al.*, 2023 ▸), materials science (*e.g.* Racioppi *et al.*, 2020 ▸; Sorbara *et al.*, 2022 ▸; Montisci *et al.*, 2023 ▸: Stachowicz *et al.*, 2023 ▸), structure–property correlation (*e.g.* Tchoń *et al.*, 2021 ▸; Gajda *et al.*, 2020 ▸, 2023 ▸; Milašinović *et al.*, 2021 ▸; Molčanov *et al.*, 2019 ▸; Stanić *et al.*, 2023 ▸; Patten *et al.*, 2023 ▸), QSAR (*e.g.* Fahimi & Matta, 2022 ▸; Vigneau *et al.*, 2022 ▸), *etc*.

However, not only experimental electron densities, but also refined X-ray restrained wavefunctions were profitably used in recent studies to extract meaningful chemical and physical properties. Just above it has been already mentioned that the XRW approach was exploited to shed further light on the effects of pressure on bis-carbonyl-annulene (Casati *et al.*, 2016 ▸, 2017 ▸). In the next paragraphs it will be also reported how the Jayatilaka technique has been recently used in chemical bonding analyses.

**Figure 16:**
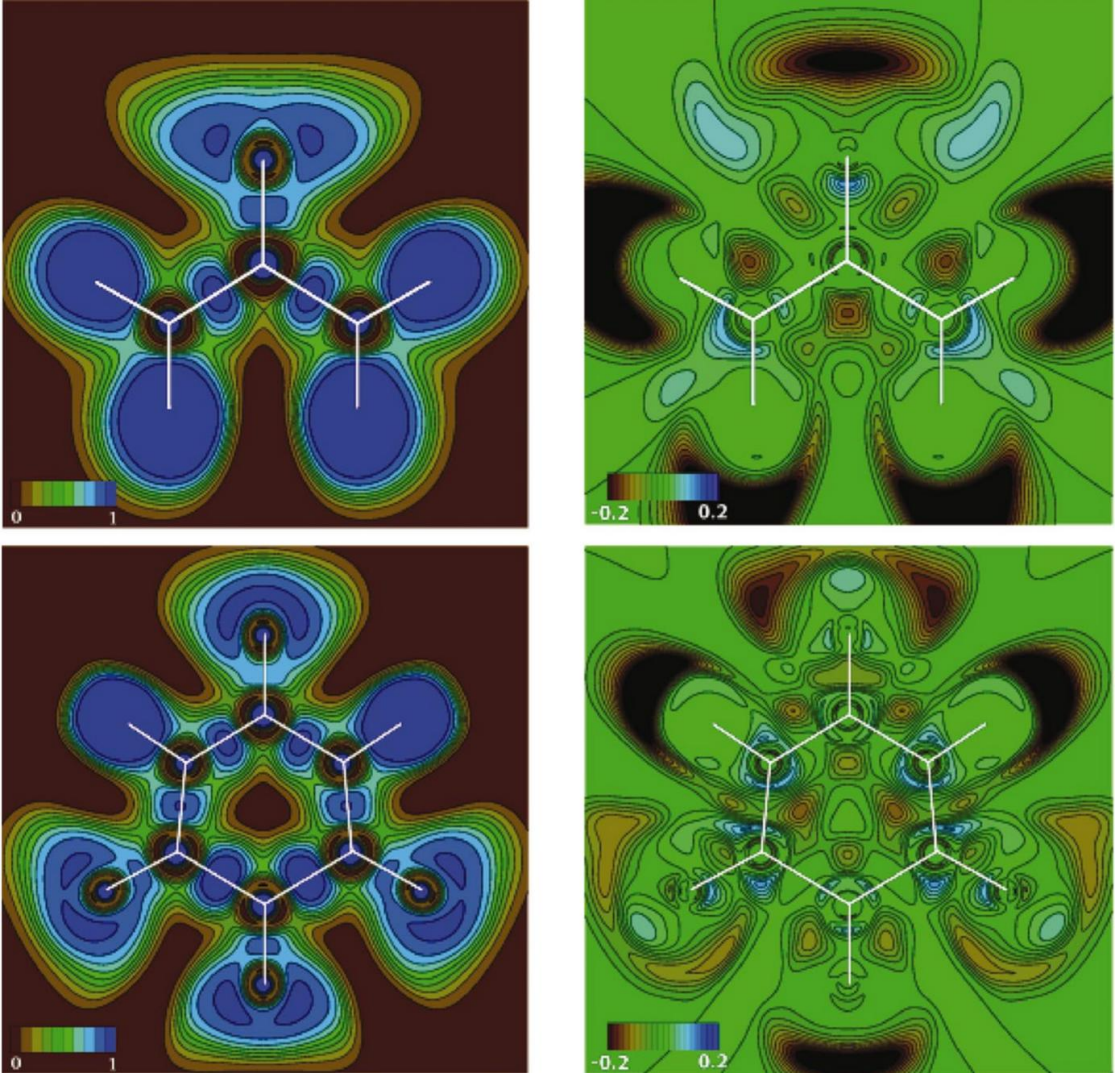
Plots of (left panels) the ELFs associated with X-ray restrained Hartree–Fock wavefunctions (contours at 0.1 increments), (right panels) the differences between the ELFs corresponding to X-ray restrained and gas-phase Hartree–Fock wavefunctions (contours at 0.02 increments), for (top panels) urea and (bottom panels) alloxan. Adapted and reproduced with permission of the International Union if Crystallography from Jayatilaka & Grimwood (2004 ▸).

A pioneering example in this direction is certainly the work by Jayatilaka & Grimwood (2004 ▸), who determined X-ray restrained wavefunctions for different molecular crystals and afterwards calculated the corresponding electron localization functions (ELFs) to show clear differences compared to the gas phase pictures (see Fig. 16 ▸). Along this line, Grabowsky and collaborators have afterwards extensively taken advantage of the XRW method to get further insights into peculiar chemical bonding situations. For instance, after the refinement of single Slater determinant wavefunctions against high-resolution X-ray diffraction data, electron localizability indicator domains were computed to explain the effects of substituents and of the crystal environment on a series of acceptor-substituted epoxide compounds (Grabowsky *et al.*, 2010 ▸). An analogous strategy was also adopted to unravel the chemistry and reactivity of α,β-unsaturated carbonyl and hydrazone systems (Grabowsky *et al.*, 2011 ▸).

Furthermore, and more interestingly, Grabowsky and coworkers have also recently published a series of XRW-based works that aimed to rationalize the problem of hypervalency. The focus was initially on sulfur dioxide (SO_2_) and on the sulfonyl group of an organic molecule (Grabowsky *et al.*, 2012 ▸). In those cases, by coupling the XRW technique with various bonding descriptors (namely, delocalization index, Roby bond index, and electron localizability indicator) and the more traditional QTAIM analysis, consensus bond orders of about 1.5 and 1.2 were obtained for SO_2_ and the sulfonyl group, respectively, thus indicating an ionic and multi-centre character for the S—O bonds. This allowed the authors to exclude hypervalency and to ascribe the shortening of the S—O bonds to the electrostatic forces associated with the ionicity of the examined systems. Following the same scheme of XRW-based analysis, the investigation of hypervalency has been later extended to phosphate, sulfate and perchlorate anions (Fugel, Malaspina *et al.*, 2019 ▸). As in the previous study, hypervalency of phosphorus and sulfur atoms was ruled out for the examined P—O and S—O bonds, while it was considered plausible for the chlorine atom and interpreted as result of the hyperconjugation between the *p*-type oxygen lone pairs and the σ* molecular orbitals corresponding to the Cl—O bonds. Along the same research line, the XRW approach was also used in combination with HAR for X-ray wavefunction refinements in two other studies: (i) in the investigation of resonance in nitric acid and in the nitrate anion (Fugel, Kleemiss *et al.*, 2018 ▸); (ii) in the complementary bonding analysis of the N—Si *peri*-interaction in two naphthyl-based pentacoordinated silicon compounds (Fugel, Ponomarenko *et al.*, 2019 ▸), where it was observed that the investigated interaction is influenced by the presence of an additional methylene group in one of the two systems and could be profitably tuned in the future by varying the fluorine atom with a series of other substituents.

To conclude this overview on the use of the Jayatilaka technique for chemical bonding analyses, it is also worth citing recent studies conducted by Thomas and collaborators. In one case, as a follow-up investigation of a multipole model charge density analysis (Thomas, Satheeshkumar *et al.*, 2015 ▸), Singh *et al.* (2024 ▸) studied the intramolecular Se—N and Se—C bonds in ebselen, an organoselenium antidepressant candidate. Based on the Roby–Gould bond orders extracted from the computed X-ray restrained wavefunction, it was possible to conclude that the Se—N bond is predominantly ionic while the Se—C bond is mainly covalent. This confirmed the hypothesis of an antioxidant activity for this class of molecules, whose drug action involves a Se—N bond cleavage. In another application, the XRW strategy was exploited for the characterization of the intramolecular S⋯O chalcogen bond in acetazolamide, collecting quite substantial evidence on the covalent nature of the examined interaction (Thomas, Jayatilaka & Guru-Row, 2015 ▸).

Finally, it is not to be forgotten that the Jayatilaka technique was also used to extract optoelectronic properties from high-resolution X-ray diffraction data. In this regard, Jayatilaka and collaborators performed XRW computations obtaining reliable polarizabilities and refractive indices (Whitten *et al.*, 2006 ▸; Jayatilaka *et al.*, 2009 ▸). They also showed that the use of multipole model electron densities unavoidably leads to much larger discrepancies from reference values for properties that need to account for at least two-body contributions in the evaluation of the quantum mechanical expectation values (Whitten *et al.*, 2006 ▸). In a later investigation, the XRW method was applied on a series of compounds characterized by non-negligible nonlinear optical properties (Hickstein *et al.*, 2013 ▸). The authors observed an average statistical deviation of 20% compared to the results of unrestrained Hartree–Fock calculations, which was afterwards considered as ascribable to the capability of the Jayatilaka method in capturing the effects of the surrounding crystal environment (Cole & Hickstein, 2013 ▸).

## 6. Possible outlooks in quantum crystallography

As is evident from the previous sections, quantum crystallography is a lively research domain, characterized by both methodological developments and applications to specific quantum problems in the crystalline state. Despite the field already being quite mature, further progress is certainly expected in the coming years.

In the traditional area of the multipole model methods we foresee several advancements. The reader can certainly anticipate a continuous evolution of the database approaches in several directions. One possible route might involve the consideration of polarization effects to better describe the interaction energies between interacting molecules, particularly in the protein-ligand cases. So far, only preliminary studies have been reported (Leduc *et al.*, 2019 ▸), focusing only on atomic polarizabilities taken from atomic basin integrations but completely disregarding bond properties and charge translation effects. One must also not forget that the polarizability tensor is better transferable when defined for a functional group, rather than for an atom (Ernst *et al.*, 2019 ▸). Therefore, the replacement of the atom-based description of polarizability with a functional group approach should be considered in the future. In addition, a comprehensive evaluation of the application of local symmetry constraints, especially in the case of the ELMAM2 database, to atomic polarizabilities and an investigation of the influence of the intramolecular dipole coupling are regarded as crucial. Furthermore, in contrast to the QTAIM partition, the anticipated enhancement of induction energies and the resulting distinct decomposition of molecular polarizabilities into atomic contributions will necessitate thorough investigations. Finally, it will be necessary to suppress the intramolecular dipole coupling with a suitable function able to simultaneously modify the polarizability decomposition into atomic contributions. This will also be of utmost importance for the definition of functional group polarizabilities within the framework of the Hansen–Coppens multipole model.

Recent technological advancements in microED, better understanding of data processing, and exploitation of the approach based on transferable multipoles (Gruza *et al.*, 2020 ▸; Kulik *et al.*, 2022 ▸) also open new perspectives to better describe electron density distributions of nanoscale crystalline materials. One could also expect more and more joint studies using simultaneously microED and X-ray diffraction data.

Last but not least, the Hansen–Coppens multipole model is expected to be further exploited for charge density determinations under extreme conditions (particularly at high pressure or for electronically excited states). For instance, since the pioneering investigation by Macchi and coworkers on BCA (see Section 5; Casati *et al.*, 2016 ▸, 2017 ▸), only a few other studies have aimed at determining experimental electron density distributions at high pressure (*e.g.* Milašinović *et al.*, 2021 ▸; Stachowicz *et al.*, 2023 ▸). However, now the potential is there and the tools are already available. Therefore, without any doubt, further outstanding studies in this research area are expected very soon.

In the framework of the wavefunction-based approaches HAR is now a well established technique, and more and more studies based on this kind of refinement strategy are expected in the future. Nevertheless, notwithstanding this high level of maturity, further methodological improvements are also envisaged. First, the extension of HAR to periodic *ab initio* calculations should be more and more developed after the pioneering work by Ruth *et al.* (2022 ▸). Furthermore, the very recent interface of HAR with the NoMoRe approach (Woińska *et al.*, 2024 ▸) will have to be further investigated and refined to perform more and more solid refinements through which both structural and thermal parameters are determined on the same quantum mechanical basis. Finally, the recently introduced concept of THAM (Chodkiewicz *et al.*, 2024 ▸) will be certainly further developed, thus leading to the construction of new libraries of Hirshfeld atom densities for fast and accurate HAR-based structural refinements.

Pertaining to the XRW method, several methodological advances and applications are also envisaged within the next decade. Among them it is worth mentioning: (i) the final solution of the long-standing problem regarding the determination of the optimal value for the λ parameter (Genoni, 2022 ▸); (ii) as for HAR, the improvement of the technique by considering periodic wavefunctions, which will enable to overcome the current limitation of the XRW strategy that can be applied only to molecular crystals; (iii) the exploitation of the XRW method to extract plausible X-ray-based exchange-correlation potentials, which could be afterwards used in the development of novel DFT exchange-correlation functionals based for the first time on experimental electron densities; (iv) the extension of the technique to other types of diffraction data (*e.g.* polarized neutron diffraction or Compton scattering data), also using simultaneously multiple types of them as already done in the multipole-model-based (Deutsch *et al.*, 2012 ▸, 2014 ▸) and density-matrix-based (Gillet, 2007 ▸; Guedidda *et al.*, 2018*a* ▸,*b* ▸) joint refinements; (v) the clarification on the reproducibility and uncertainties of the XRW computations, as suggested by recent publications (Landeros-Rivera *et al.*, 2021 ▸; Davidson *et al.*, 2022*b* ▸; Bürgi & Genoni, 2022 ▸); (vi) and finally the improvement of the X-ray wavefunction refinement (Woińska *et al.*, 2017 ▸; Davidson *et al.*, 2022*a* ▸), with an alternation of the HAR and XRW techniques until convergence in both structural and electronic parameters.

In the field of the quantum chemical topology approaches, a finer tuning and improvement of the already developed techniques are expected. For example, it would be desirable the extension of the spin density source function to the analysis of experimental spin distributions obtained through multipole model joint refinements. Along the same line, one could also envisage the use of the IQA technique along with experimental electron densities resulting from traditional multipole model refinements or X-ray restrained wavefunction calculations. Furthermore, concerning the *PolaBer* strategy, a natural next step is to include the influence of an oscillating electric field, thus introducing frequency-dependent polarizabilities. Among many possible schemes, the one introduced by Seidler *et al.* (2016 ▸) seems to be the most promising and has already proven to be accurate when predicting linear optical properties. Dynamic polarizabilities will enhance the utility of benchmarking database-derived polarizabilities against experimentally observed parameters.

As is clear from the previous lines, quantum crystallography is characterized by very large variety of methods whose number is going to inevitably increase in the next years. In this context, a desirable strategy would consist in regrouping the programs associated with the different techniques in a common platform that would eventually serve not only as a complete software package for quantum crystallographic studies, but also as a starting point for future methodological developments. This is the direction currently being explored by Coles, Puschmann and Ruth in the development of the promising *Quantum Box* project that should hopefully become the reference for future investigations in quantum crystallography.

Finally, to conclude this review, it is also worth noting that, apart from the above-mentioned future perspectives, collaborations with other research areas outside quantum crystallography are also very likely and will be always very welcomed. For instance, in the next years, one could imagine interactions with scientists working in the field of single-molecule diffraction through free-electron laser experiments (Odate *et al.*, 2023 ▸). Although this research will not be strictly related to the domain of problems in the crystalline state, collaborations in this direction could be very fruitful to get further insights into the analyses of chemical bonds. It is also possible to envisage the development of novel quantum crystallographic techniques that will take advantage of modern computational technologies, such as artificial intelligence and quantum computing. In these directions, promising and encouraging examples are the already mentioned machine learning-based strategies proposed in the framework of quantum chemical topology (Burn & Popelier, 2020 ▸, 2022 ▸, 2023 ▸; Isamura & Popelier, 2023 ▸; Gallegos *et al.*, 2022 ▸; Gallegos & Martín Pendás, 2023 ▸) and the very recent and pioneering study conducted by Rahm and coworkers (Skogh *et al.*, 2023 ▸), who reported the first topological analyses of molecular electron densities obtained through current quantum computers. More studies along these lines are expected and will be gladly received by the whole quantum crystallographic community.

All in all, after more than a century since its birth, quantum crystallography continues to be a vibrant field of scientific inquiry offering many interesting research perspectives for the decades to come. We eagerly look forward to seeing its further developments in the coming years.
